# A Novel Sushi-IL15-PD1 CAR-NK92 Cell Line With Enhanced and PD-L1 Targeted Cytotoxicity Against Pancreatic Cancer Cells

**DOI:** 10.3389/fonc.2022.726985

**Published:** 2022-03-22

**Authors:** Da-Lai Xu, Yuan-Qing He, Bin Xiao, Yuan Si, Jian Shi, Xue-Ang Liu, Lei Tian, Qian Ren, Ya-Song Wu, Yi Zhu

**Affiliations:** ^1^ Pancreas Center, The First Affiliated Hospital of Nanjing Medical University, Nanjing, China; ^2^ Pancreas Institute of Nanjing Medical University, Nanjing, China; ^3^ Department of General Surgery, The First Affiliated Hospital of Nanjing Medical University, Nanjing, China; ^4^ Laboratory Animal Research Center, Jiangsu University, Zhenjiang, China; ^5^ Research & Development Department, Timmune Biotech Inc., Tianjin, China

**Keywords:** pancreatic ductal adenocarcinoma, Chimeric antigen receptor (CAR), IL-15, IL-15Rα-sushi, programmed cell death ligand 1 (PD-L1), adoptive immunotherapy

## Abstract

Pancreatic ductal adenocarcinoma (PDAC) is a highly aggressive and lethal malignancy with a limited response to current therapies. Novel and effective treatment is urgently needed. Herein, a chimeric antigen receptor (CAR)-NK92 cell line, with an interleukin (IL)-15Rα-sushi/IL-15 complex and a Programmed cell death-1(PD1) signal inverter was constructed and named SP ( ***S*** ushi-IL15- ***P*** D1). We showed that CAR expression enabled SP cells to proliferate independently of IL-2 and became more resistant to nutrition starvation-induced apoptosis. Meanwhile, SP cells were more effective than NK92 in PDAC cell killing assays *in vitro* and *in vivo*, and there was a positive correlation between the killing capability of SP cells and PD-L1 expression in pancreatic cancer cells. Based on the synergistic and comprehensive effects of the special CAR structure, the adhesion, responsiveness, degranulation efficiency, targeted delivery of cytotoxic granule content, and cytotoxicity of SP cells were significantly stronger than those of NK92. In conclusion, the SP cell line is a promising adoptive immunotherapy cell line and has potential value as an adjuvant treatment for pancreatic cancer, especially in patients with high PD-L1 expression.

## Introduction

Pancreatic cancer is a highly malignant disease having a 5-year survival rate of less than 10%, with pancreatic ductal adenocarcinoma (PDAC) accounting for more than 90% of all pancreatic cancers (1, 2). PDAC has proven to be extremely difficult to manage due to its rapid-growth characteristics, high relapse rate even after radical resection, and resistance to nearly all conventional therapies (3–5). None of the highly anticipated immune checkpoint inhibitors (ICI), including antibody drugs targeting programmed cell death 1 (PD-1), programmed cell death ligand 1 (PD-L1), or cytotoxic T lymphocyte associated protein 4 (CTLA-4), have demonstrated significant effects on PDAC (6, 7). Therefore, new effective therapy to improve PDAC prognosis is urgently needed.

Recently, the potential role of natural killer cells (NK cells) in tumor immunotherapy has been recognized (8). NK cells respond quickly to transformed and compressed cells (9) and have the inherent potential to engraft into almost all human tissues. NK cells recognition of target cells is independent of MHC- and tumor-associated antigen (10), which makes allotransplantation and targeting cancer cells that evade T-cell killing by reducing MHC molecule expression possible. NK cells do not secrete large amounts of IL-6, which reduces the risk of severe cytokine storms (11, 12). Nonetheless, NK cells only account for approximately 10% of blood cells; thus, a tedious process of blood cell separation and enrichment is needed. The low transfection efficiency also makes gene modification of NK cells more difficult.

Apart from primary NK cells, the NK cell lymphoma patient-derived NK cell line NK92 can also be used in adoptive immunotherapy (13). NK92 cells have shown cytotoxic activity against a wide range of tumor cells (14–17) and are amenable in large-scale cultures and gene manipulation. Moreover, their characteristic for low side effects has already been demonstrated in several clinical trials (18, 19).

Chimeric antigen receptor (CAR) engineering is currently a hot topic in the field of immune oncology. CD19-CAR T cell therapy has achieved a striking complete remission rate of more than 80% in refractory or relapse (R/R) acute lymphoblastic leukemia (20) and 40%–58% in R/R B cell lymphoma (21, 22). NK92 cells can also be genetically engineered to produce effector cell lines, such as CAR-engineered NK-92 cells (23–26).

Several studies, including meta-analyses, have found that PD-L1-positive or high expression of PD-L1 in PDAC patients displayed evidence of lymphocyte exhaustion, and was associated with more advanced TNM stage, and poor prognosis (27–29). Therefore, we attempted to construct a PD-1/PD-L1 signal inverter using NK92 cells to reverse the inhibitory signals from PD-L1 in pancreatic cancer cells to activation signals with strong targeted cytotoxicity toward PD-L1 positive tumor cells.

The design strategy of the CAR in this study was a construct based on the combination of the extracellular domain of the PD1 molecule, which recognizes and binds its ligand PD-L1, an IL-15Rα-sushi/IL-15 complex ligated with the CD8 transmembrane domain, 4-1BB and CD3ζ domain, and their expression in NK92 cells using lentiviral vectors. The IL-15Rα-sushi/IL-15 complex offers SP cells a proliferative advantage and enhanced cytotoxicity; thus, a stronger killer cell line was created. The PD-1 containing CAR structure could transmit the inhibitory signal of PD-L1 into activation by linking the PD-1 extracellular domain into the “BBz” domain widely used in CAR-T activation, therefore a “PD1 negative signal inverter” was installed. Based on the key components in the CAR, the established CAR-NK92 cell line was named SP (** *S*** ushi-IL15-** *P*** D1).

Thus, the present study aimed to verify the killing effect of the novel adoptive immunotherapy cell line SP cell line on PDAC cells, and to analyze the main mechanisms allowing SP to exert a stronger killing effect. The expansion advantages of SP cells, which are crucial to clinical application, were investigated in terms of their proliferative ability, changes in cell cycle dynamics, and apoptosis while the *in vitro* cytotoxic activity of SP cells was tested against PDAC cells harboring different levels of PD-L1. Finally, a peritoneal tumor-bearing mouse model of human PDAC cells was constructed to investigate the *in vivo* anti-tumor effects.

## Materials and Methods

### Cell Culture

NK92 cells were maintained in α-MEM (Invitrogen) medium containing 12.5% fetal bovine serum (FBS) (Invitrogen), 12.5% horse serum (HyClone), 2 mM L-glutamate, and supplemented with 100 U/mL rhIL-2 (Changchun Institute of Biological Products Ministry of Public Health People’s Republic of China). The Human PDAC cell lines (MIA PaCa-2, CFPAC-1, SW1990) were cultured in DMEM (Gibco, USA) supplemented with 10% FBS, 100 U/mL penicillin, and 100 μg/mL streptomycin. Cell cultures were maintained at 37°C in a humidified atmosphere containing 5% CO_2_.

### Establishment of the SP CAR-NK92 Cell Line

We designed cDNA fragments of a chimeric antigen receptor (CAR) encoding signal peptide, IL-15Rα-sushi, transmembrane domain of CD8, signal transduction zone of 4-1BB, and CD3ζ, 2A signal peptide, PD1 extracellular region, and IL-15 series, with restriction sites *BamHI* and *EcoRI* present in the multiple clone site of the cloning vector and lentiviral vector. After base codon optimization, the full-length DNA sequence was synthesized and cloned into a pCMV-Blank vector (Beyotime, China, # D2602) to obtain pCMV-CAR. Then, the CAR fragment was ligated with pLVX-M-puro (Addgene, USA, #125839) to obtain the pLVX-CAR plasmid.

To generate viral particles, the Lenti-X™ 293T cell line (Takara Bio companies, #632180) was cultured overnight to 70%–80% density in a 150-mm plate for transfection. The plasmid pCMV-VSV-G (Addgene, #8454), pCMV-dR8.2 dvpr (Addgene #8455), and pLVX-CAR were mixed at a mass ratio of 1:3:4 to formulate the co-transfected plasmid (40 µg). A 120 µg Polyethyleneimine solution (Sigma, Cat# GF70215825) was added dropwise to obtain the plasmid-PEI mixture. The plasmid-PEI mixture was incubated at room temperature for 30 min and transfected into 293T cells. The transfected cells were cultured overnight at 37°C in a CO_2_ incubator for 8-16 h. Next, the cell culture medium was discarded, and a 20 mL volume 10% FBS DMEM was added together with sodium butyrate to obtain a final concentration of 10 mM. The supernatant of the cell culture was collected after incubation at 37°C for 48 h with CO_2_. The supernatant was centrifuged for 5 min at 1500 rpm and filtered through a 0.45 µm filter. The virus solution was added at a mass percentage of 40% PEG solution by 1/3 volume, mixed, and placed overnight at 4°C. The samples were centrifuged at 2000 ×g at 4°C for 45 min the next day. After the supernatant was discarded, the virus precipitate was resuspended in MEM-alpha medium with 1/10 volume of virus stock solution to obtain a 10-fold concentration of virus suspension.

Purified and concentrated lentiviruses were used to transfect NK92 cells. Approximately 3×10^5^ NK92 were resuspended with 1 mL virus and 8 µg/mL polybrene (Sigma, H9268-5G) to augment infection efficiency; cells were was transferred to a 24-well plate. Three hours after infection, the supernatant was removed and the NK92 culture medium containing IL-2 at a final concentration of 100U/mL was added for the amplification culture. The next day, after the infection was repeated, NK92 culture medium containing IL-2 at a final concentration of 100U/mL, and the culture medium was maintained for 3 days until passage. After 2 weeks, IL-2 was no longer added to the culture medium, and cultures in IL-2-free medium were continued for another 2 weeks. This novel ***S*** ushi-IL15-** *P*** D1 CAR-expressing NK92 cell line (SP) with stable expression of the CAR gene was obtained by the finite dilution method. The IL-2-free medium was GT-T551-H3 medium (TAKARA, Cat#WK593S) and contained the serum substitute (HELIOS, Cat# HPCFDCGL50) with a final concentration of 2.5% (volume percentage).

All gene fragments encoded by the CAR construct were expressed in tandem a single CMV promoter. Please see **Supplemental File Data Sheet 1** for detail of amino acid numbering for elements used to CAR.

### Lentivirus Transfection

Lentiviruses expressing the luciferase gene were purchased from Shanghai Genechem Co., Ltd. (Shanghai, China). Lentiviruses expressing PD-L1 and the luciferase gene were purchased from Obio Technology (Shanghai, China). Three strains of PDAC cells were inoculated into the plate in advance, and when the cell density reached 20%, the virus and the corresponding transfection enhancer were added according to the manufacturer’s instructions. After 12 h, the culture medium was replaced. When the cells grew to 80% density, puromycin was added to screen cells expressing the luciferase gene or PD-L1 gene, and the surviving cells were continuously cultured with halved puromycin concentrations. The expression of luciferase was verified using a bioluminescence imaging assay. The expression of PD-L1 was verified using flow cytometry (FCM).

### CCK-8 Assay

Cell proliferation was investigated using the Cell Counting Kit-8 assay (CCK-8) (Dojindo, China) according to the manufacturer’s instructions. Briefly, cells were seeded in 96-well plates at a density of 3×10^3^ cells/100 μL/well. After 1-, 2-, 3-, or 4-day culture, 10 μL of CCK-8 reagent was added to each well. The plates were incubated in the dark at 37°C for 1 h, and then the absorbance at 450 nm was measured. For the long-term proliferation experiment, the CCK-8 assay was performed repeatedly at 15-day intervals, and the cell amplification ratio was measured at a 72-hour period.

### 5-Ethynyl-2’-Deoxyuridine Assay

The 5-ethynyl-2’-deoxyuridine assay (EdU) assay was performed according to the manufacturer’s instructions (YF^®^488/555/594/647A Click-iT EdU Imaging Kits, US Everbright^®^ Inc., Suzhou, China). Briefly, after a 2-hour incubation with EdU, the cells were fixed with 4% paraformaldehyde and permeabilized with 0.5% Triton-X. After Click-iT reaction buffer treatment, cell nuclei were stained with Hoechst 33342. Images were obtained using a high-content imaging system (ImageXpress Micro 4, Molecular Devices, Shanghai, China), and cell proliferation was assessed as the percentage of EdU-positive cells.

### Cell Cycle Analysis

Cells were fixed in 70% ethanol at 4°C for 5 h and then washed twice with 1× binding buffer. The cells were then stained with 4 μL RedNucleus I in 1 mL PBS for 20 min at 37°C (Cell Cycle Assay Kit Plus, US Everbright^®^ Inc., Suzhou, China). Stained cells were assessed by FCM (CytExpert, Beckman Coulter, USA) and analyzed using ModFit LT5.0.

### Apoptosis Detection

Cells were cultured in PBS for 8 h and then suspended in 100 μL Annexin V binding buffer (YF^®^488-Annexin V and PI Apoptosis Kit, US Everbright^®^ Inc., Suzhou, China), stained with 5 μL Annexin V and 5 μL PI and incubated for 15 min in the dark at 4°C. Cells were harvested and analyzed by FCM.

### Cytotoxicity Assay

Pancreatic cells expressing the luciferase gene were seeded in 96-well plates (1×10^4^ cells/100 μL/well), and after 6 h of culture in complete medium, NK or SP cells were added with different effector to target (E:T) ratios. The total amount of volume per well was maintained at 200 μL. After a 12-h co-culture, 50 μL 0.1% Triton X-100 was added to each well and mixed. After a 5 min rest, 50 μL of the supernatant was collected and transferred to the black 96-well plate, 50 μL light-emitting substrate mixture (2 mg/mL ATP [Aladdin, Shanghai, China] and 300 ng/mL D-luciferin [BioVision, USA] at a ratio of 1:3) were added to each well. The emissions were detected using the *In-Vivo* Xtreme Imaging System (Bruker, Germany) within 5 min. The percentage of cell lysis was calculated using the standard formula: 100%×(control photons–experimental photons)/control photons.

### Xenograft Mouse Model

For *in vivo* experiments, two strains of cells with different expression levels of PD-L1 were used to construct a mouse model, namely the parental MIA PaCa-2-Luc and the MIA PaCa-2 transfected with PD-L1 (named PD-L1^high^MP-Luc). To establish intraperitoneal dissemination PDAC xenograft mouse models, 4–6 week-old female BALB/c nude mice purchased from Cavens (Changzhou, China) were injected i.p. with 2×10^6^ MIA PaCa-2-Luc or PD-L1^high^MP-Luc cells suspended in 200 μL PBS under phenobarbital anesthesia on day 0. Injected mice were randomly assigned to three treatment groups as follows: the PBS controls, NK92 cells, and SP cells. Approximately, 5.5×10^6^ irradiated NK92 or SP cells (10 Gy, 200 cGy/min) were intravenously infused at 3 intervals from day 5 to day 26, for a total of 8 injections. Equal volumes of PBS were infused in parallel to the PBS control group. All mice were observed following an i.p. injection of 150 mg/kg D-luciferin potassium (Shanghai Sciencelight Biology Science & Technology Co., Shanghai, China) at 6 timepoints from day 1 to day 19 using the *In-vivo* Xtreme Imaging System to detect tumor growth. Overall survival was monitored and recorded. All animal experiments were reviewed and approved by the Institutional Animal Care and Use Committee of Jiangsu University.

### Carboxyfluorescein Diacetate Succinimidyl Ester Staining

Cells were washed and suspended in 1mL of PBS at a density of 2×10^6^/mL, and then incubated at 37°C with 2 μM carboxyfluorescein diacetate succinimidyl ester (CFSE) (Sigma) for 10 min. Quench staining was performed on ice for 5 min by adding 5 volumes of ice-cold DMEM with 10% FBS. The cells were then washed three times with ice-cold PBS containing 1% bovine serum albumin and cultured under appropriate conditions.

### Conjugate-Formation Detection

NK92 or SP cells were incubated with CFSE-labeled target cells at an effector to target ratio of 1:1 for 0, 10, or 20 min. The cell mixture was then gently suspended and fixed with 2% paraformaldehyde. The cell mixture was stained with anti-CD56-APC antibody at 4°C for 15 min and analyzed by FCM. The percentage of conjugate was calculated as CFSE and APC double-positive events (30).

### Integrin (LFA-1) Assays

To evaluate the level of LFA-1 in open conformation, NK92 or SP cells were mixed with target cells at an E:T ratio of 5:1, centrifuged at 300 ×g for 1 min, incubated at 37°C for 15 min, and then stained with anti-CD56-APC and anti-LFA-1-PE antibody clones TS1/22, specific for the open, high-affinity conformation of LFA-1 (31) at 4°C for 15 min. The proportion of PE-positive APC-positively gated cells was analyzed by FCM.

### Cell Surface Marker Detection

To evaluate the PD-L1 level in PDAC cells, cells were incubated with PE-labeled anti-human PD-L1 antibody at 4°C for 15 min, washed twice, and analyzed by FCM. Similarly, antibodies against CD56, Fas-L, NKG2D, NKp30, CD226, TRAIL, PD1, LFA-1, and IL-15 were used to evaluate their expression in NK92 and SP cells. Detailed antibody information is listed in **Supplementary Table 1**.

### Degranulation and Cytokine Production Assay

NK92 or SP cells labeled with anti-CD107a-BV421 antibody were incubated with target cells at a 1:1 ratio at 37°C for 6 h. Brefeldin A and monensin (Thermo Fisher) were added 1 h after the beginning of incubation, while anti-IFN-γ-PC5.5 and anti-TNF-α-APC antibodies were added after fixation and permeabilization. Assessment of CD107a, IFN-γ, and TNF by FCM was performed as previously described (32).

### Live Cell Staining and Analysis

NK cells were incubated with Lyso-Tracker Red (Beyotime Biotechnology, China), a red fluorescent probe specific to lysosomes. Target cells were stained with calcein-AM (Dojindo, China), a green fluorescent probe to label live cells. Before co-culture, the fluorescence of effector and target cells were inspected using a high-content imaging system. Next, effector cells were added into the wells containing target cells seeded for 4 h in advance (E:T = 5:1), and the plate was immediately visualized using a high-content imaging system, red fluorescence detected by Cy5 channel, green fluorescence detected by FITC channel, and time-lapse imaging was performed at 1-minute intervals for 90 min.

After co-culture, the suspension effector cells were gently separated from the adherent target cells. Lyso-Tracker Red labeled red fluorescence was detected by both FCM and using a high-content imaging system. The reduction rate of mean fluorescence intensity (MFI) of red fluorescence was calculated using the formula: 100%×(“MFI of red fluorescence before co-culture” - “MFI of red fluorescence after co-culture”)/”MFI of red before co-culture”. Target cells were inspected using a high-content imaging system for calcein-AM-labeled green fluorescence, as well as the red fluorescence adopted from effector cells. The reduction rate of MFI of green fluorescence was calculated using the formula: 100%×(“MFI of green fluorescence before co-culture” - “MFI of green fluorescence after co-culture”)/”MFI of green fluorescence before co-culture”. Data were obtained from five replicate wells.

### Statistics

Data are presented as the mean ± SEM. SPSS software (version 20.0, SPSS Inc., Chicago, IL, USA) was used for the statistical analysis. For normally distributed endpoints, one-way analysis of variance was used for three or more independent group comparisons. For the cell proliferation CCK-8 assay and *in vivo* bioluminescence intensity, repeated-measures analysis of variance was used. Survival curves were plotted according to the Kaplan-Meier method and compared using the log rank test. Statistical significance was set at *P* < 0.05.

## Results

### Generation of Sushi-IL15-PD1 CAR Expressed NK92 Cells

**Figure 1:**
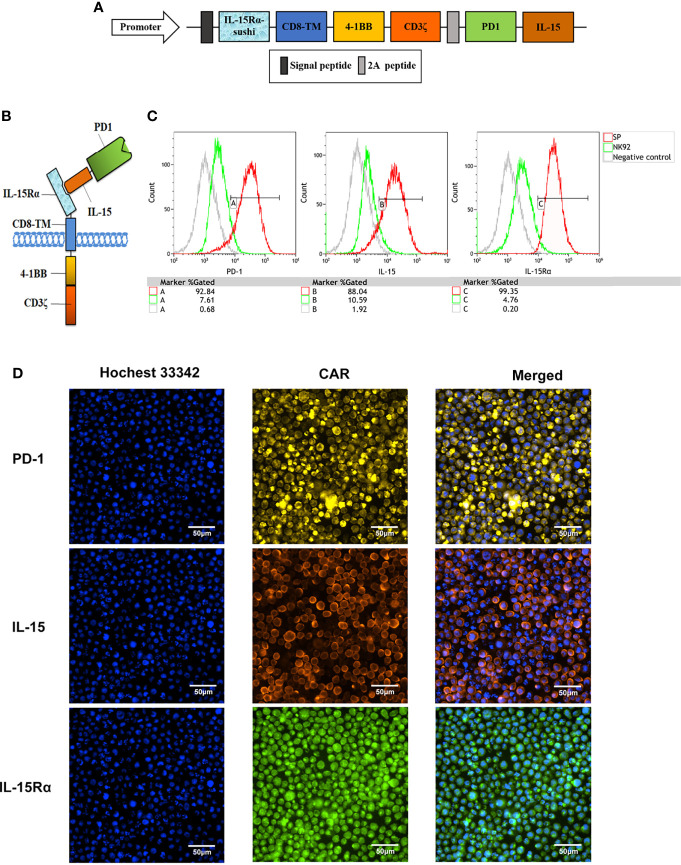
Establishment of a novel sushi-IL15-PD1 CAR-NK92 cell line (SP). **(A)** Sequential expression cassette diagram of the chimeric antigen receptor (CAR), including signal peptide, IL-15Rα-sushi, CD8 transmembrane domain, signal transduction domain of 4-1BB and CD3ζ, 2A peptide, PD1 extracellular domain and IL-15. **(B)** Structure diagram of the protein expression pattern of the CAR gene. **(C)** Surface expression of programmed cell death 1 (PD-1), IL-15Rα, and IL-15 in SP and NK92 cells was analyzed by flow cytometry (FCM). **(D)** Cellular immunofluorescence detection of CAR molecules localized and expressed on SP cell membrane.

The structure of the protein complex and the nucleic acid sequence corresponding to the protein structure of the chimeric antigen receptor of CAR-NK92 (SP), respectively, are shown in **Figures 1A, B** and in **Supplementary Figure 1**. Compared with parental NK92 cells, FCM demonstrated that SP cells highly expressed protein molecules PD1 and IL-15 with immunological activity. The positive rates for PD1 were 92.84% and 7.61%, for IL-15 were 88.04% and 10.59%, for IL-15Rα were 99.35% and 4.76%, for SP and NK92 cells, respectively (**Figure 1C** **)**. Cellular immunofluorescence verified that the structures of CAR molecules were highly expressed on SP cell membranes (**Figure 1D**). After the CAR gene was transcribed under the control of the strong CMV promoter, the translated proteins of each domain component could be correctly folded and stably expressed. According to NK92 is dependent on cytokines for survival, only the NK92 cells stably transfected with CAR gene continued to proliferate and passage in medium without cytokines, demonstrating that the SP cell line had been established successfully.

### SP Cells Proliferated Independently of Exogenous IL-2 and Became More Resistant to Nutrition Starvation-Induced Apoptosis

**Figure 2:**
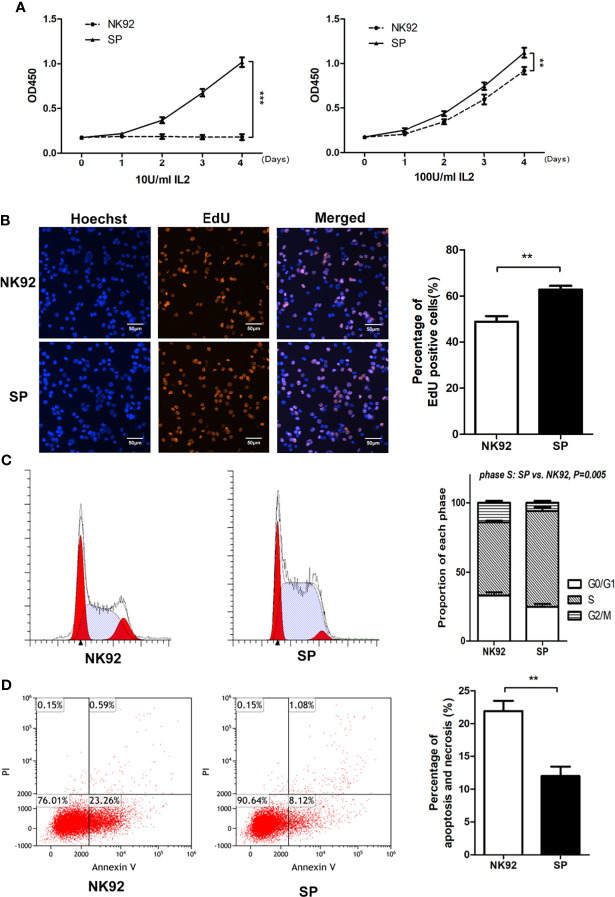
The new CAR-NK-92 (SP) cell line could be stably passaged and expanded independently of exogenous IL-2. **(A)** Short term proliferation of SP and NK92 cells exposed to 10 U/mL IL-2(left) and 100 U/mL IL-2 (right) supplements. **(B)** 5-Ethynyl-2’-deoxyuridine (EdU) assay and **(C)** cell cycle analysis were performed using SP cells and NK92 cells cultured with the 100 U/mL IL-2 supplement. **(D)** Shown is the percentage of apoptotic cells was detected by FCM after culturing NK92 and SP cells in PBS for 8 hours. The left sides of panels **(B–D)** show a representative sample image of the experimental results, and the right side shows the statistical results of three parallel experiments expressed as mean ± SEM (n = 3, **P < 0.01, ***P < 0.001).

Microscopic observation showed that compared with NK92 cells, the SP cell clones were larger, brighter, and plumper (**Supplementary Figure 2A**). We used the CCK-8 assay to quantitatively detect the proliferative ability of the two cell lines. SP cells could maintain sustained proliferation under low IL-2 (10 U/mL) supplementation, while NK92 cells could not. In the standard IL-2 condition (100 U/mL), both NK92 and SP cells proliferated stably, while SP cells showed a higher growth rate (*P =* 0.002, **Figure 2A**). In the standard IL-2 (100 U/mL) culture conditions, the percentage of EdU-positive cells in the SP group was significantly higher than that in the NK92 group (*P =* 0.008). Furthermore, the proportion of cells in the S phase was also significantly higher in the SP group than in the NK92 group *(P =* 0.005, **Figures 2B, C** **)**.

SP cells were cultured in complete medium without IL-2, while NK92 cells were cultured in complete medium supplemented with 100 U/mL IL-2. The 3-day amplification times were detected every 15 days and monitored continuously for 3 months. Compared with the NK92 group supplemented with 100 U/mL IL-2, the SP group without IL-2 had stronger proliferation ability and was more stable (*P = 0.002*, **Supplementary Figure 2B**).

NK92 or SP cells cultured in PBS were used to simulate nutrition starvation conditions. The proportion of apoptotic cells in the SP group was significantly lower than that in the NK92 group (*P =* 0.009) (**Figure 2D**), demonstrating that SP cells may have adopted resistance against apoptosis or nutrition starvation.

Taken together, these results were consistent and showed that compared with NK92 cells, SP cells were characterized by “IL-2 independence and nutrient starvation tolerance” and thus presented an advantage for clinical grade large-scale cell expansion, which supported the expression and function of the IL-15Rα-sushi/IL-15 complex in the CAR structure of SP.

### SP Cells Exerted Higher Cytotoxicity Against PDAC and Positively Correlated With the Expression of PD-L1 in PDAC

**Figure 3:**
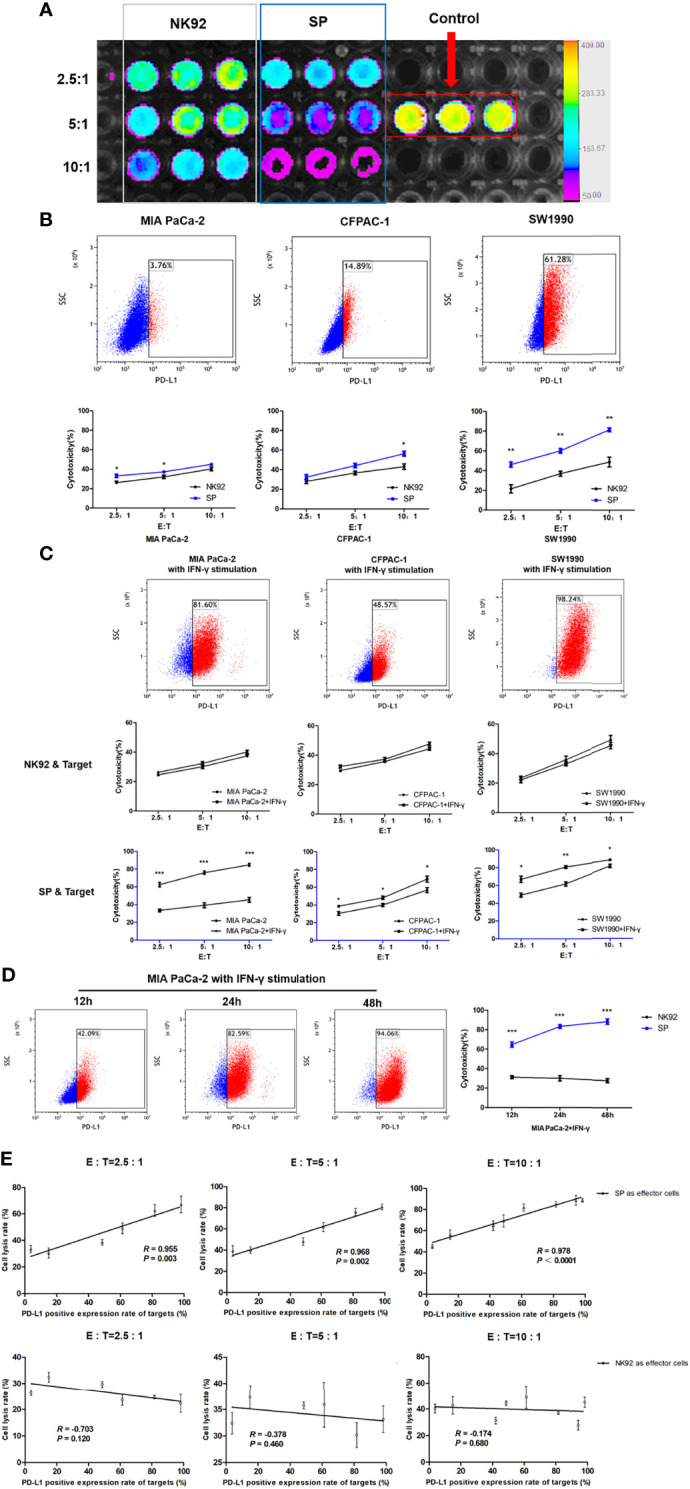
Enhanced cytotoxicity of SP cells against both innate and IFN-γ-induced PD-L1-positive PDAC cells in a PD-L1 dose-dependent manner. **(A)** Cytotoxicity of SP or NK92 cells against SW1990 cells was measured by bioluminescence imaging after a 12-hour co-culture. **(B)** Expression of programmed cell death ligand 1(PD-L1) in three Pancreatic ductal adenocarcinoma (PDAC) cell lines (MIA PaCa-2, CFPAC-1, SW1990) was detected by FCM, and the lysis rate of SP and NK92 cells against each cell line was analyzed. **(C)** The three PDAC cell lines were stimulated with 5 ng/mL IFN-γ for 24 hours, the expression of PD-L1 in each cell line and the cytotoxicity of SP or NK92 cells against each cell line were detected. **(D)** MIA PaCa-2 cells were stimulated with 5 ng/mL IFN-γ for 12, 24, and 48 hours, to induce the expression of PD-L1 and the cytotoxicity achieved by SP or NK92 cells in co-cultures were assessed. **(E)** A correlation analysis between the cytotoxicity and target cell PD-L1 level. All summary data are indicated as mean ± SEM (n = 3). *P < 0.05, **P < 0.01 and ***P < 0.001.

Three PDAC cell lines, MIA PaCa-2, CFPAC-1, and SW1990 were used in this study. MIA PaCa-2 is a poorly differentiated stem-like PDAC cell line isolated from undifferentiated human pancreatic cancer (33). CFPAC-1 is a moderately differentiated PDAC cell line established from the liver metastases of PDAC. SW1990 is a well-differentiated cell line established from splenic metastatic foci of PDAC and is also considered to be gemcitabine resistant (34). The cytotoxicity of SP or NK92 cells co-cultured with PDAC cells was quantitatively detected using the luciferase assays (**Figure 3A**). The expression of PD-L1 in MIA PaCa-2, CFPAC-1, and SW1990 was 3.76%, 14.89%, and 61.28%, respectively (**Figure 3B**). The cell lysis rate increased with a higher effector-target ratio for both NK92 and SP, while the cytotoxicity of SP was stronger than that of NK92 at any given effector-target ratio (**Figure 3B**). The cell lysis rate of SP was higher than that of NK92 in all three target cell lines, and for SW1990, having the highest PD-L1 expression, the difference was shown to be the largest, indicating PD-L1-dependent cytotoxicity (**Figure 3B**).

Imai et al. (35) reported that IFN-γ could stimulate PD-L1 expression in PDAC cells. In our experiment, we also confirmed that all three PDAC cell lines cultured in medium containing IFN-γ showed higher PD-L1 levels (**Figure 3C**). As shown in our cytotoxic assays, the cytotoxicity of NK92 was not significantly affected by the target cell PD-L1 level, while SP cells exerted significantly higher killing efficiency when confronted with target cells with higher PD-L1 levels (**Figure 3C**). Among the three target cells, MIA PaCa-2 cells were most sensitive to IFN-γ stimulation, with a proportion of PD-L1 expression of 3.76%, 42.09%, 82.59%, and 94.06% at 0, 12, 24, and 48 h of IFN-γ stimulation, respectively. SP cell cytotoxicity was also significantly elevated (*P* < 0.001) as the target cell expression of PD-L1 increased, compared to the cytotoxicity of NK92 cells **(** **Figure 3D**).

Based on the data of our repeated cytotoxic assays, we performed a comprehensive correlation analysis between cell lysis rate and PD-L1 level, and the results showed a significant positive correlation for SP cells (*R* = 0.955, *R* = 0.968, *R* = 0.97 with corresponding *P*-values: *P* = 0.003, *P* = 0.002, *P* < 0.001, at different E:T ratios: 2.5:1, 5:1, 10:1, respectively), which was not observed for NK92 cells (**Figure 3E**).

Taken together, the above results demonstrated that SP cells adopted a selective and sensitive killing capability against PD-L1 positive target cells, indicating that SP cells had the ability to invert the inhibitory PD-L1 signal into an activation and killing signal.

#### *In Vivo* Tumor Inhibition and Prolonged Survival of SP Cells Treated PDAC Xenograft Mouse Model

To construct tumor-bearing mouse models with different PD-L1 expression levels, we selected MIA PaCa-2 cells with 5.85% PD-L1 expression for transfection of the PD-L1 gene. FCM verified that the expression of PD-L1 in MIA PaCa-2 cells after transfection (named PD-L1^high^MP-Luc) reached 98.79% (**Supplementary Figure 3A**). Before undergoing *in vivo* treatment, we tested the effects of γ-irradiation on the growth and cytotoxicity of NK92 and SP cells. After exposure to either 5 or 10 Gy γ-irradiation, further replication of both NK92 and SP cells was prevented (**Supplementary Figure 3B**). The use of 10 Gy γ-irradiation could ensure the effective survival of NK92 and SP for only 3 days. The cytotoxicity assay of effector cells on PD-L1^high^MP-Luc showed that cytotoxicity was not affected by irradiation (**Supplementary Figure 3C**), and compared with the NK92 group, the SP group still maintained higher cytotoxicity (**Supplementary Figure 3D**).

**Figure 4:**
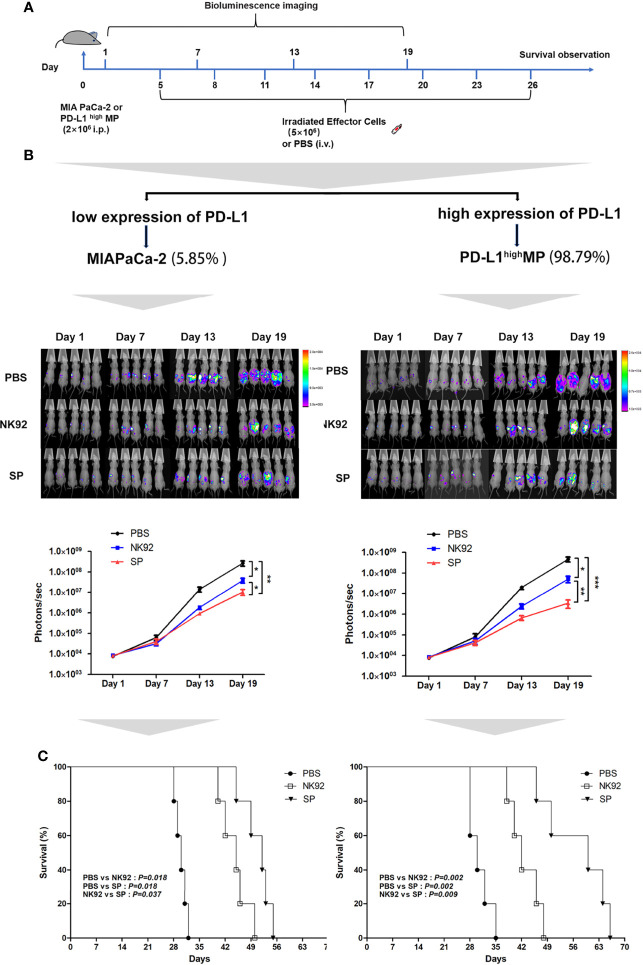
*In vivo* anti-tumor efficiency of SP cells in MIA PaCa-2 and PD-L1^high^MP xenograft mouse model. **(A)** The construction of mouse model, and treatment protocol and observation. **(B)** Bioluminescence imaging was used to observe the photons of mouse abdominal cavity tumors reflecting the tumor size, to evaluate the therapeutic effects of SP and NK92 cells on abdominal tumor-bearing mice, as well as the statistical results of the photon levels. The left side shows the experimental results of the wild-type strain MIA PaCa-2 tumor-bearing mice, and the right side shows the experimental results of the PD-L1^high^MP tumor-bearing mice stably transfected with the PD-L1 gene. Summary data are shown as the mean ± SEM, (*P < 0.05, **P < 0.01). **(C)** Overall survival was monitored and analyzed using Kaplan-Meier method. ***P < 0.001.

Female nude mice aged 4–6 weeks were intraperitoneally injected with MIA PaCa-2-Luc cells (PD-L1 low-expressing strain) or stably transfected lentivirus overexpressing PD-L1 (PD-L1^high^MP-Luc) cells on day 0 and were randomly allocated to the three treatment groups: PBS, NK92 cells, and SP cells. Irradiated NK92 or SP cells were infused intravenously on days 5, 8, 11, 14, 17, 20, 23, and 26, respectively. Tumor burden was assessed by bioluminescence imaging at 6-day intervals (**Figure 4A**).

**Figure 4B** shows the detection of tumors in mice, and **Figure 4C** shows the overall survival of mice. The left side of **Figures 4B, C** are the results of three different treatment groups on MIA PaCa-2-Luc tumor-bearing mice, and the right side shows the results of three different treatment groups on PD-L1^high^MP-Luc tumor-bearing mice.

The results of adoptive immunotherapy of MIA PaCa-2-Luc tumor-bearing mice showed that compared with the PBS treatment group, both the NK92 and SP treatment groups could effectively inhibit tumor growth in mice (NK92 vs. PBS: *P* = 0.017, SP vs. PBS: *P* = 0.009), although, the SP treatment group showed stronger inhibitory effects than the NK92 treatment group (SP vs. NK 92: *P* = 0.032) (**Figure 4B** **left)**. The median survival times of the PBS treatment group, NK92, and SP treatment groups were 30, 45, and 52 days, respectively. The overall survival of the SP treatment group was higher than that of the NK92 group (SP vs. NK92: *P* = 0.037, **Figure 4C** **left**).

The results of adoptive immunotherapy of PD-L1^high^MP-Luc tumor-bearing mice with high PD-L1 expression showed that compared with the other two treatment groups, the treatment effect of SP was significantly enhanced (SP vs. PBS: *P <* 0.001; SP vs. NK 92: *P* = 0.010) **(** **Figure 4B** **right**). The median survival times of the PBS, NK92, and SP treatment groups were 30, 42, and 60 days, respectively and the overall survival of mice in the SP treatment group was significantly prolonged (SP vs. PBS: *P* = 0.002; SP vs. NK92: *P* = 0.009; **Figure 4C** **right**).

In summary, the above results indicated that compared with NK92 cells, SP cells had a significantly stronger ability to inhibit tumor growth *in vivo* than NK92 cells, and could significantly extend the survival of tumor-bearing mice. Targeting PD-L1^high^MP-Luc tumor-bearing mice, the therapeutic effect of SP was more significant than that of NK92 and PBS treatment groups.

### Mechanisms Underlying the Enhanced Cytotoxicity

#### Main Mechanisms Used by SP to Exert Stronger Killing Effects on PDAC Cells

*In vitro* and *in vivo* experiments involving the co-culture of SP cells (NK92 as control group) and PD-L1^high^MP cells were used to systematically investigate the main mechanisms underlying the enhanced SP cell cytotoxicity.

**Figure 5:**
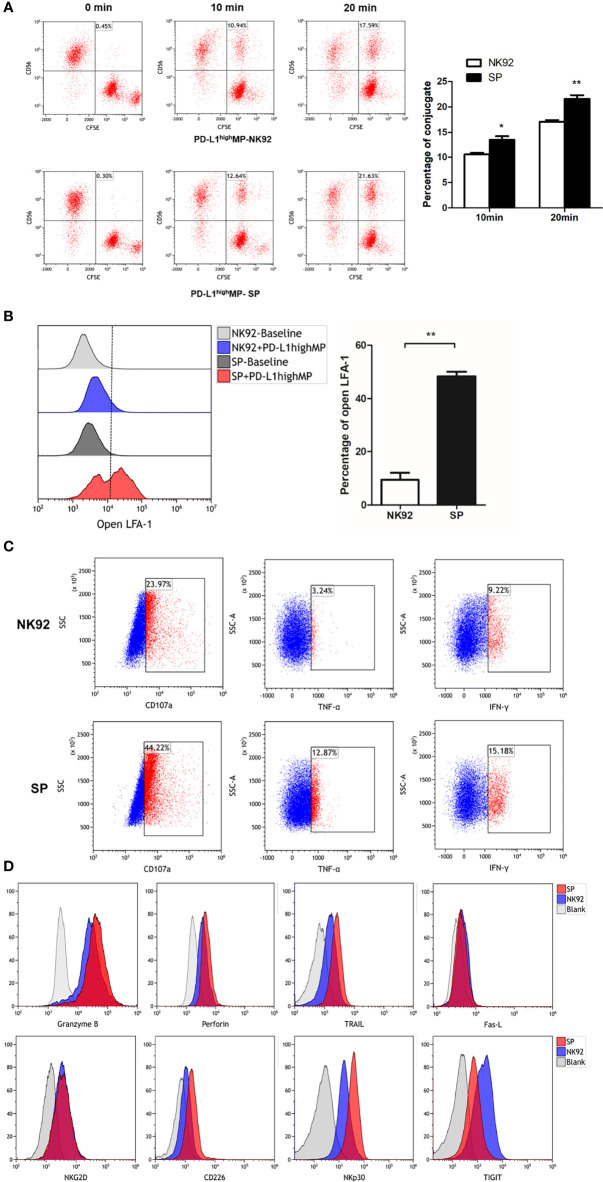
FCM detection of SP cells conjugate formation, degranulation and cell death signaling transduction. **(A)** SP or NK92 cells were incubated with CFSE-labeled PD-L1^high^MP cells and assessed for the proportion of conjugate formation (CFSE and CD56 double positive counts). **(B)** SP or NK92 cells were assessed for the expression of LFA-1 in open-conformation following incubation with PD-L1^high^MP. **(C)** CD107a, TNF-α and IFN-γ expression of SP or NK92 cells were detected after a 6-hour incubation with PD-L1^high^MP. **(D)** SP or NK92 cells were assessed for expression of markers including granzyme B, perforin, TRAIL, Fas-L, NKG2D, CD226, NKp30, and TIGIT using FCM. For panels A and B, dummary data are shown as mean ± SEM (*P < 0.05, **P < 0.01, n = 3).

The process of NK cell killing involves a series of reactions, including conjugate/immune synapse formation, degranulation/signal transduction, and programmed cell death (36). Conjugate formation has been proven to be a prerequisite for NK cell cytotoxicity (30); thus, we compared the effector-target cell co-localization ratio using NK92 or SP cell-mediated cytotoxic assays. PD-L1^high^MP cells were labeled with CFSE, and NK92, or SP cells were labeled with anti-CD56-APC antibody, and the proportion of double positive components was investigated through FCM after 10 or 20 min of co-culture. Our data showed that SP cells tended to have a higher co-localization ratio than NK92 cells at both 10 min (10.6% ± 0.5% vs. 13.5 ± 1.2%, *P* = 0.017) and 20 min (17.1% ± 0.6% vs. 21.6% ± 1.3%, *P* = 0.005) culture periods, demonstrating that SP cells presented a higher conjugate formation capacity (**Figure 5A**).

The open conformation of the integrin molecule LFA-1 (CD11a) was reported to be positively related to adhesion and conjugate formation (37). We used an antibody specific to LFA-1 in an open conformation to evaluate our findings from this perspective. NK92 or SP cells were incubated with PD-L1^high^MP for 15 min, and the proportion of open LFA-1 was detected by FCM (**Figure 5B**). The percentage of open LFA-1 in SP was significantly higher than that in NK92 (48.3% ± 3.1% vs. 9.4% ± 4.5%, *P* < 0.001), thus confirming the enhanced conjugate formation activity of SP.

Second, CD107a is a marker for degranulation; TNF-α and IFN-γ are cytokines representative of signal transduction in response to target cells, and we utilized antibodies specific for these three markers to stain control NK92 or SP cells after 6 h of stimulation by PD-L1^high^MP cells. The FCM results showed that the positive rates of all three markers in SP cells were higher than those in NK92 cells (44.22% vs. 23.97% for CD107a, 12.87% vs. 3.24% for TNF-α, and 15.18% vs. 9.22% for IFN-γ) **(** **Figure 5C**).

Third, NK92 cells induce apoptosis (or programmed cell death) mainly through two pathways. One is the activated NK cell pathway which releases particles, such as secretory lysosomes containing perforin or granzyme B, which in turn fuse with target cells and induce apoptosis (36). Another apoptosis-inducing pathway involves activated NK cell-expression of Fas-L and TRAIL (TNF-related apoptosis-inducing ligand), which activate the apoptosis signaling cascade through Fas or TRAIL receptors on the target cell surface. Thus, we compared the expression of perforin, granzyme B, Fas-L, and TRAIL between SP and NK92 cells. Our results showed that TRAIL expression was dramatically higher in SP cells (57.3% vs. 16.2%, **Figure 5D**), while perforin and granzyme B expression was only slightly higher (MFI: 3159 vs. 1944, 35067 vs. 22430, respectively), while no differences were observed in Fas-L expression.

We also evaluated the expression of classical phenotypic molecules related to cytotoxicity in SP and NK92 cell lines (**Figure 5D**). The results showed that the expression of CD226/DNAM-1 and NKp30 proteins was higher in SP than in NK92 cells, and the positive rates of CD226/DNAM-1 and NKp30 in SP vs. NK92 were 59.4% vs. 16.2% and 84.1% vs. 48.0%, respectively. The positive rate of TIGIT expression was decreased in SP vs. NK92 (60.1% vs. 87%). NKG2D expression did not differ, and other detected but non-differentially expressed markers included CD16, CD57, KIR, and NKp46 (data not shown).

#### Visualization of the Degranulation of Effectors in the Co-Culture of Effectors and Targets

**Figure 6:**
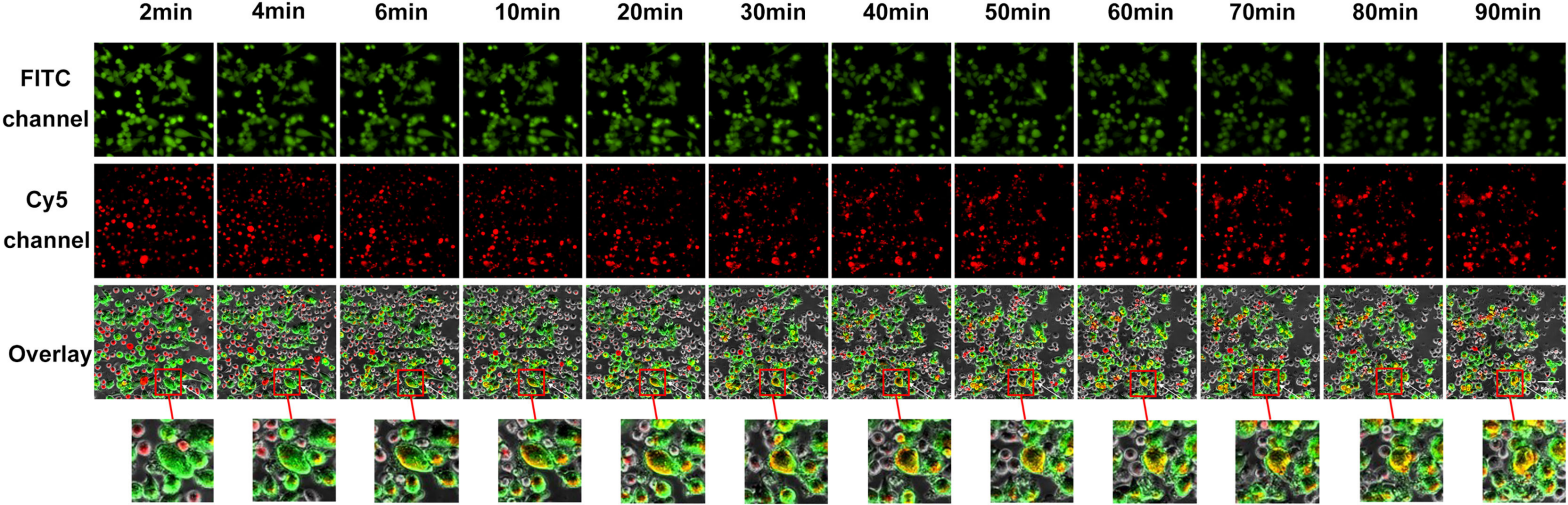
Visual and dynamical observation of the delivery process of secretory lysosomes of SP cells to PD-L1^high^MP cells. From left to right are the images from 12 sequential time points of the effect-target cell co-culture experiment. From top to bottom, the first row indicates fields of view under the green fluorescence (FITC) channel, the second row indicates fields of view under the red fluorescence (Cy5) channel, and the third row shows the overlay of the two-fluorescence signals. Dynamically changes can be observed following the co-culture of effector and target cells, and the red box indicates typical degranulation phenomenon, in which SP cells delivered secretory lysosomes labeled by Lyso-Tracker Red fluorescent probe to PD-L1^high^MP cells.

The exocytosis of secretory lysosomes (degranulation) is a key process in NK cell-mediated cytotoxicity (38). To investigate lysosome delivery, we stained lysosomes in NK92 or SP cells with Lyso-Tracker Red, and stained target PD-L1^high^MP cells with Calcein-AM, which is a probe for specific green fluorescence labeling of living cells. Target cells were seeded in advance, and effector cells were added after target cell adhesion, and time-lapse imaging was utilized to record the degranulation of effectors in the co-culture of effector cells and target cells using a high-content live cell imaging system. Images of 12 sequential time points were captured and analyzed (**Figure 6** and **Supplementary Figure 4**).

#### Greater Efficiency of Degranulation and Killing Capability of SP Cells Was Quantified by High Content Imaging System Combined With FCM

We discovered that the degranulation-exocytosis process of NK cells could be monitored in real-time as well as by combining a high-content imaging system and FCM, and the efficiency of lysosome particle transport from effector cells to target cells and killing capability could be quantitatively measured.

**Figure 7:**
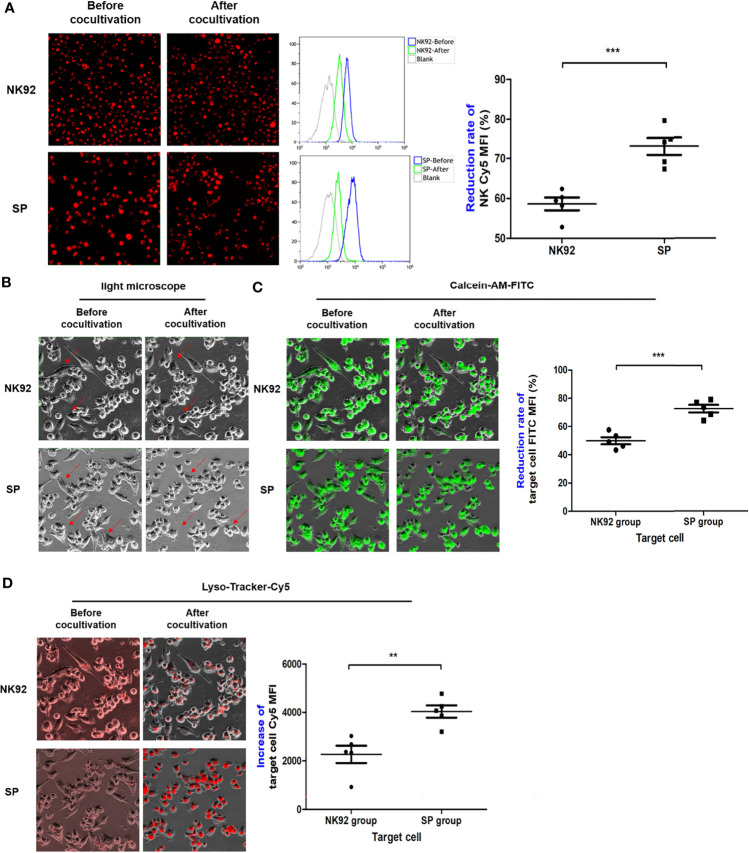
The degranulation of SP and the targeted delivery of cytotoxic granule content were more efficient and lethal. **(A)** After co-culture, effector cells were collected and the changes in red fluorescence were detected by FCM, indicating the lysosome exocytosis. Morphological changes in target cells before and after co-culture were observed in bright field **(B)**, while FITC channel for **(C)** quantitative assessment of lysosomes entering target cells as indicated by increased red fluorescence mean fluorescence intensity (MFI) (Cy5 channel). **(D)** Target cells death as indicated by green fluorescence (MFI FITC decrease). All summary data showed mean ± SEM, n = 5 different fields. **P < 0.01, ***P < 0.001.

For suspension effector cells, as shown in **Figure 7A**, the MFI of red fluorescence after co-culture detected by the Cy5 channel of the high-content imaging system and FCM was decreased compared to before co-culture. Thus, measuring the rate of decline in red fluorescence of effector cells, the rate of MFI decline of SP cells was significantly greater than that of NK92 cells (74.1% ± 2.2% vs. 58.6% ± 1.6%, *P* < 0.001), which indicated enhanced secretory lysosome exocytosis and higher degranulation efficiency.

For adherent target cells, the fluorescence intensity was quantitatively detected by a high-content imaging system using a bright field, both the FITC channel for green fluorescence, or Cy5 channel for red fluorescence were used for comparative evaluation. In the bright field observation, the morphology of a portion of the adherent PDAC cells clearly changed from a fusiform to round shape, and the changes were more abundant and obvious in SP co-cultures (**Figure 7B**). In the FITC channel, the MFI of target cells of both the NK92 and SP cell co-cultures was reduced, and the reduction rate of the SP group was significantly higher than that in the NK92 group (72.7 ± 2.7% vs. 50.0 ± 2.5%, *P* < 0.001, **Figure 7C**). Thus, both bright field and green fluorescence data of target cells supported the higher killing capability of SP cells. In the Cy5 channel, the MFI values of target cells from both NK92 and SP cell co-culture also increased, and the increased MFI range of target cells from the SP group was significantly higher than that obtained in the NK92 group (4363.3 ± 257 vs. 2270.0 ± 358, *P* = 0.004, **Figure 7D**), providing further evidence for the stronger lysosomal delivery efficiency of SP cells.

Taken together, the above results demonstrated that, consistent with the test results of CD107a, SP had stronger capabilities for degranulation, directional delivery of cytotoxic contents, and cytotoxicity compared with NK92.

## Discussion

### Design Strategy of the SP Preparation

In this study, we designed a novel CAR construct, equipped with the classical 4-1BB & CD3ζ complex as the intracellular domain, which was comprised of a complex containing the PD1 protein and an IL-15Rα-sushi/IL-15 complex. The novel construct was stably transduced into NK92 cells to obtain a new cell line, SP. IL-2 or IL-15 are necessary for the survival and proliferation of NK and CD8+T cells (39). Although both IL-2 and IL-15 receptor complexes have unique α chains, the β and γ chains are shared. IL-15Rα-sushi (the sushi domain of the IL-15 receptor α chain) functions as a super agonist of IL-15 (40, 41). The IL-15Rα-sushi/IL-15 complex can completely replace the role of IL-2 in activating NK/CD8+T cells, and the membrane-bound expression of this complex is able to activate both the expressing cells in cis and standard NK/T cells in trans (42). Therefore, theoretically, SP cell infusion should be able to activate innate immunity in the body and exert a composite anti-tumor effect in addition to cytotoxic activity. Our new SP cell line may also switch the inhibitory signals of PD-L1 into activation signals, which has also been described in previous studies (43, 44). PD-L1 positivity or high expression of PDAC positively correlated with poor prognosis of PDAC patients in prior studies (27–29), suggesting that the novel and valuable characteristics of our SP cell line could have potential application in the treatment of PDAC.

Further, this study proved that compared with NK92, the novel SP cells did not rely on IL-2 to stably expand *in vitro*, resist apoptosis or nutrition starvation, and maintain their capacity for cytotoxicity. Thus, SP is an engineered cell line with the potential for clinical development and application in adoptive immunotherapy of tumors.

### Anti-PDAC Tumor Effect of SP *In Vitro* and *In Vivo*

A xenograft tumor-bearing mouse model was established. Previous studies have shown that, intraperitoneal inoculation of tumor cells is simple and easy to control in animal models. However, a meta-analysis evaluating the recurrence pattern of 17,313 cases of pancreatic cancer after resection showed that 13.5% of patients exhibited peritoneal spread, and intraperitoneal metastasis was a key factor affecting poor outcomes (45). According to the clinical dose of irradiation of NK92 cells (46) and the survival time of NK92 cells after irradiation, we determined that a therapeutic dose of 5×10^6^ irradiated effector cells injected through the tail vein would be required, and the treatment regimen was once every 3 days for a total of eight injections. The results of the *in vivo* experiment overlapped with those of the *in vitro* experiments, which showed that compared with the parental NK92s cell, SP not only had a stronger killing ability against PDAC cells, but also the killing ability of SP positively correlated with the level of PD-L1 expression of PDAC, indicating that SP cells gained the ability to invert the inhibitory PD-L1 signal into an activation and killing signal to target PDAC cells expressing PD-L1.

SP cells significantly inhibited the tumor growth of pancreatic cancer cells *in vivo* and prolonged the survival time of tumor-bearing mice. However, adoptive therapy with SP cells was not able to eradicate tumors in animal models completely due to the complexity of solid tumors, suggesting that further studies on the number and treatment using immune cell transfer, the modality of transfer, together with the enhancement and improvement of CAR signals are represent areas for optimization of the approach, which are crucial for the final clinical efficacy.

### Mechanisms of Enhanced SP Cell Cytotoxicity

We systematically examined the key molecules and pathways involved in the entire process of effector-target killing and statistically analyzed the differences between the SP and NK92 treatment groups using a high-content imaging system combined with FCM to achieve the technical advantages of real-time monitoring and quantitative analysis. The results of comprehensive experiments verified that: (i) the novel SP was stably transfected and expressed specific CAR structural genes in its parental NK92 cell line; (ii) Based on the specific CAR structure, the endogenous expression of open conformation LFA-1 molecule, activated the CD226/DNAM-1 receptor and NKP30 molecules, while apoptosis-inducing TRAIL molecules in SP cells were significantly increased compared with those of NK92 and the endogenous expression of the inhibitory receptor TIGIT was significantly decreased; and (iii) SP cells exhibited significantly stronger effects compared to NK92 cells in terms of adhesion, reactivity, degranulation efficiency, directional delivery of cytotoxic granule contents, and cytotoxic effect on pancreatic cancer cell lines with high PD-L1 expression. Thus, SP cells showed a significantly enhanced killing capability against PD-L1 positive pancreatic cancer cells *in vitro* and *in vivo*.

## Conclusion

**Figure 8:**
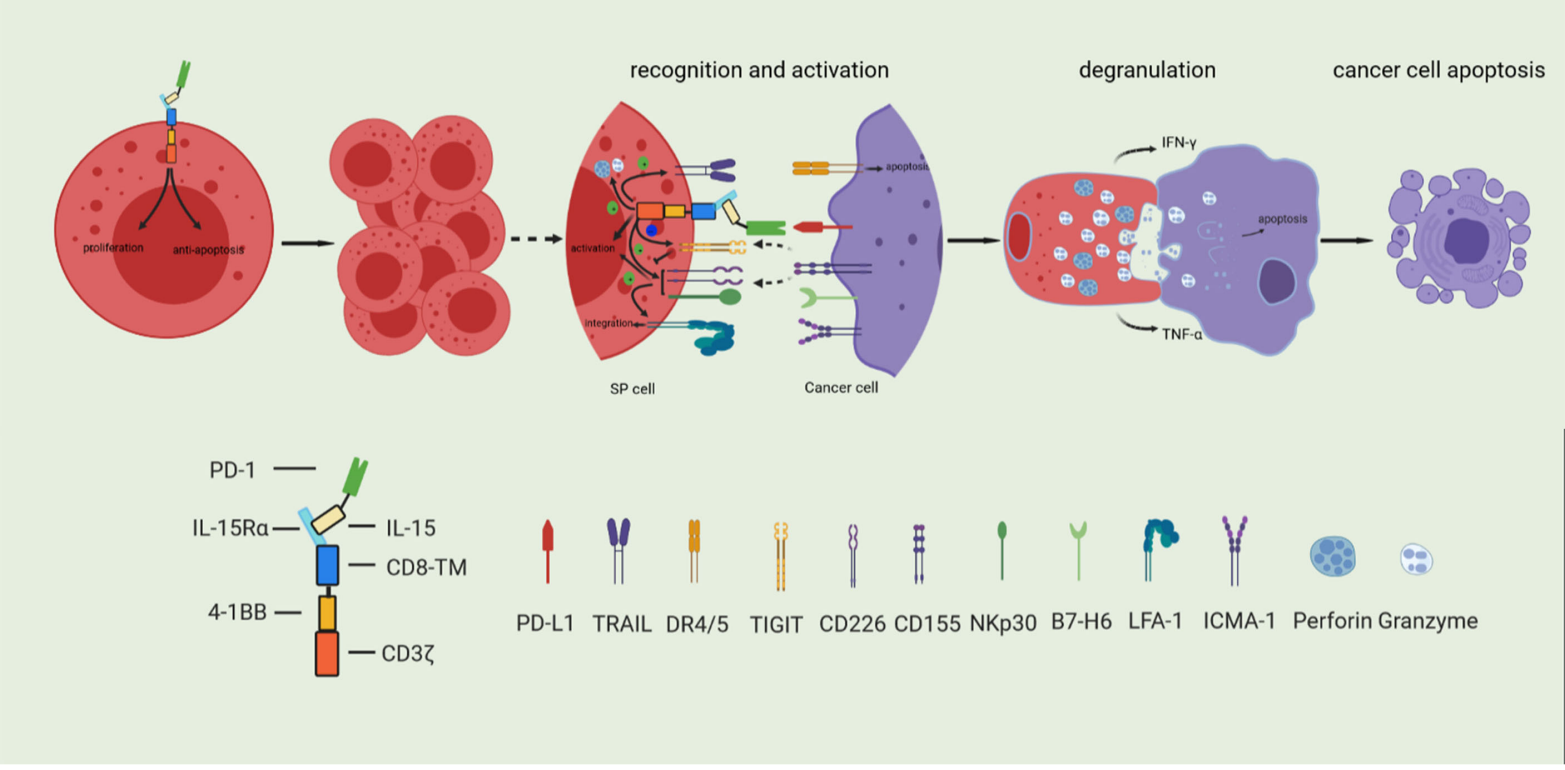
Based on the synergy and comprehensive effects of special CAR structure, SP cells have enhanced proliferative and anti-apoptosis ability, and comprehensively enhanced cytotoxicity.

We described the development of a novel ***S*** ushi-IL15-** *P*** D1 CAR-NK92 (SP) cell preparation. As shown in **Figure 8**, SP cells express the IL-15/IL-15Rα complex, which enables stable cell expansion independently of exogenous IL-2, and exhibit a stronger anti-apoptotic ability even in the context of nutritional deprivation. These characteristics are conducive to the large-scale expansion of this preparation for transfer to clinical applications with significant cost savings; SP cells also express PD1, and because of the design strategy including the PD-L1/PD1 negative signal inverter, PD-L1/PD1 immunosuppressive signals can be converted into activation signals, characterized by a strong killing capability against PD-L1 positive pancreatic cancer cells. Based on the synergy and comprehensive effects of its specific CAR structure, the adhesion, responsiveness, degranulation efficiency, targeted delivery of cytotoxic granule content, and cellular toxicity were significantly stronger than those of the parent NK92 cells. In conclusion, SP represents a promising cell line for adoptive immunotherapy and has potential value as an adjuvant treatment for PDAC, especially in patients with PDAC characterized by high PD-L1 expression.

## Data Availability Statement

The original contributions presented in the study are included in the article/**Supplementary Material**. Further inquiries can be directed to the corresponding authors.

## Ethics Statement 

The animal study was reviewed and approved by Institutional Animal Care and Use Committee of Jiangsu University.

## Author Contributions

YZ and Y-SW contributed to the conception of the study. YZ and Y-QH designed research. D-LX and BX performed research. YS and JS contributed significantly to analysis and manuscript preparation. D-LX and X-AL performed the data analyses and wrote the manuscript. LT and QR helped perform the analysis with constructive discussions. All authors contributed to the article and approved the submitted version.

## Funding

This work was supported by grants from the National Natural Science Foundation of China (81672471) and Jiangsu Key Medical Discipline (General Surgery) (ZDXKA2016005).

## Conflict of Interest

Authors YS and Y-SW were employed by Timmune Biotech Inc.

The remaining authors declare that the research was conducted in the absence of any commercial or financial relationships that could be construed as a potential conflict of interest.

## Publisher’s Note

All claims expressed in this article are solely those of the authors and do not necessarily represent those of their affiliated organizations, or those of the publisher, the editors and the reviewers. Any product that may be evaluated in this article, or claim that may be made by its manufacturer, is not guaranteed or endorsed by the publisher.

## Supplementary Material

Supplementary Figure 1 The DNA sequence of the novel sushi-IL15-PD1 CAR.

Supplementary Figure 2 Characteristics of SP cells. **(A)** The observation of NK92 cell line and SP cell line by microscopic (×100). **(B)** The SP group was cultured without IL-2, and the NK92 group was cultured with 100U/ml IL-2. The 3-day amplification multiple was detected every 15 days and monitored continuously for 3 months. The 3-day proliferation multiple of the cells in the two groups was compared and analyzed.

Supplementary Figure 3 Effector and target cell preparation before *in vivo* experiments. **(A)** The PD-L1 expression of PD-L1^high^ MP cells was detected by FCM. **(B)** Detection of the proliferation activity of NK92 and SP cells irradiated with γ-irradiation at different doses. **(C)** Detection and statistical analysis of the killing effect of NK92 and SP cells on PD-L1^high^ MP before and after 10Gy γ-irradiation (n=3, *P<0.0 5, **P<0.0 1, *P<0.001). (D) Comparation of the killing ability of NK92 cells and SP cells to PD-L1^high^ MP after irradiation.

Supplementary Figure 4 Visual and dynamical observation of the delivery process of secretory lysosomes of NK92 cells to PD-L1^high^MP cells. From left to right are the images from 12 sequential time points of the effect-target cell co-culture experiment. From top to bottom, the first row indicates fields of view under the green fluorescence (FITC) channel, the second row indicates fields of view under the red fluorescence (Cy5) channel, and the third row shows the overlay of the two-fluorescence signals. Dynamically changes can be observed following the co-culture of effector and target cells, and the red box indicates typical degranulation phenomenon, in which NK92 cells delivered secretory lysosomes labeled by Lyso-Tracker Red fluorescent probe to PD-L1^high^MP cells.

## Abbreviations

PDAC, Pancreatic ductal adenocarcinoma; SP, ***S*** ushi-IL15-** *P*** D1; FCM, flow cytometry; CAR, chimeric antigen receptor; PD-1, programmed cell death 1; PD-L1, programmed cell death ligand 1; MFI, mean fluorescence intensity.
