# Supplementary figures and images for: A Novel Sushi-IL15-PD1 CAR-NK92 Cell Line With Enhanced and PD-L1 Targeted Cytotoxicity Against Pancreatic Cancer Cells
Source: Front Oncol. 2022 Mar 22;12:726985. doi: 10.3389/fonc.2022.726985 (PMC8980464; doi:10.3389/fonc.2022.726985)

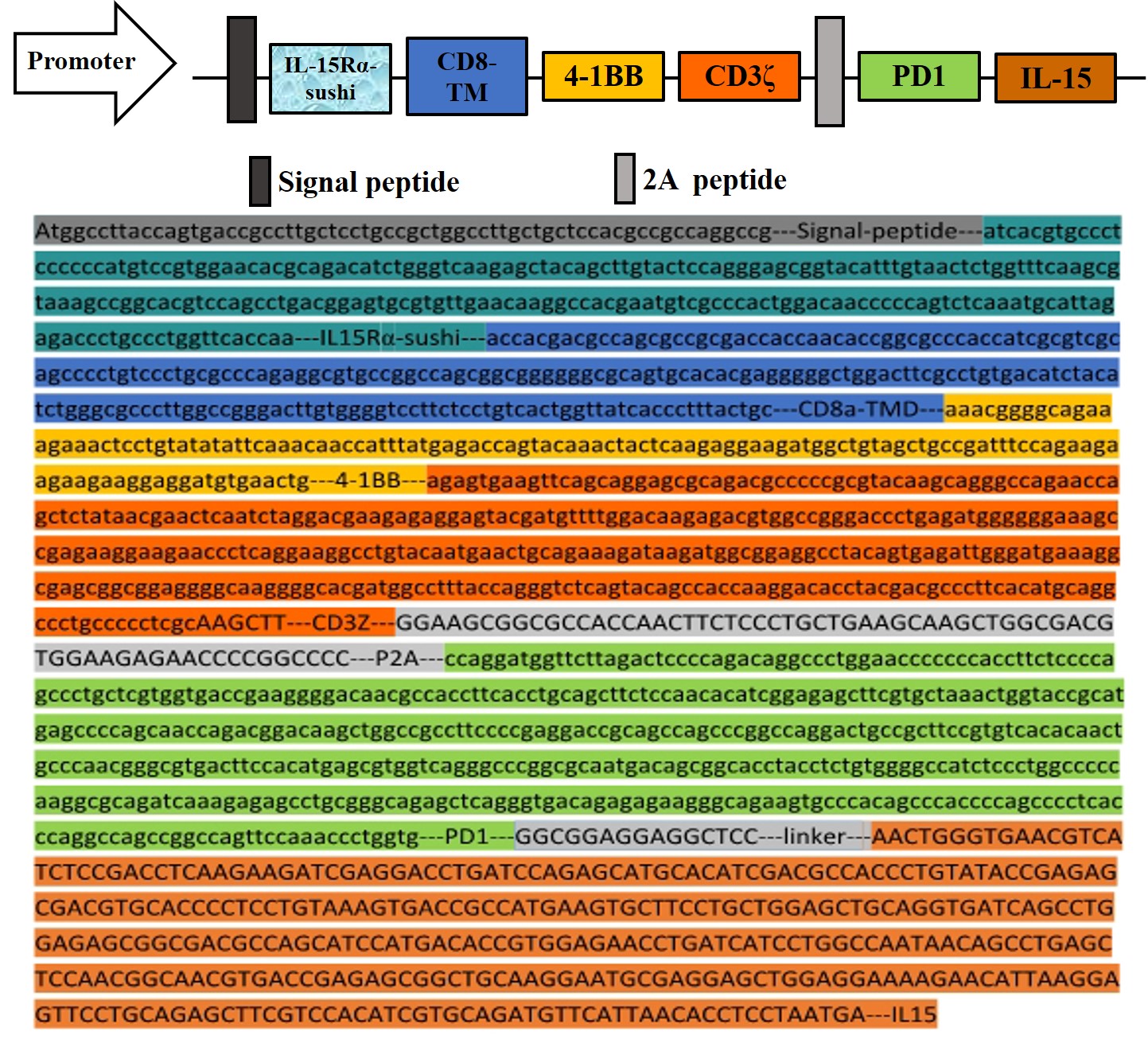

Supplement: Supplementary Figure 1 — The DNA sequence of the novel sushi-IL15-PD1 CAR. [file Image_1.jpeg]

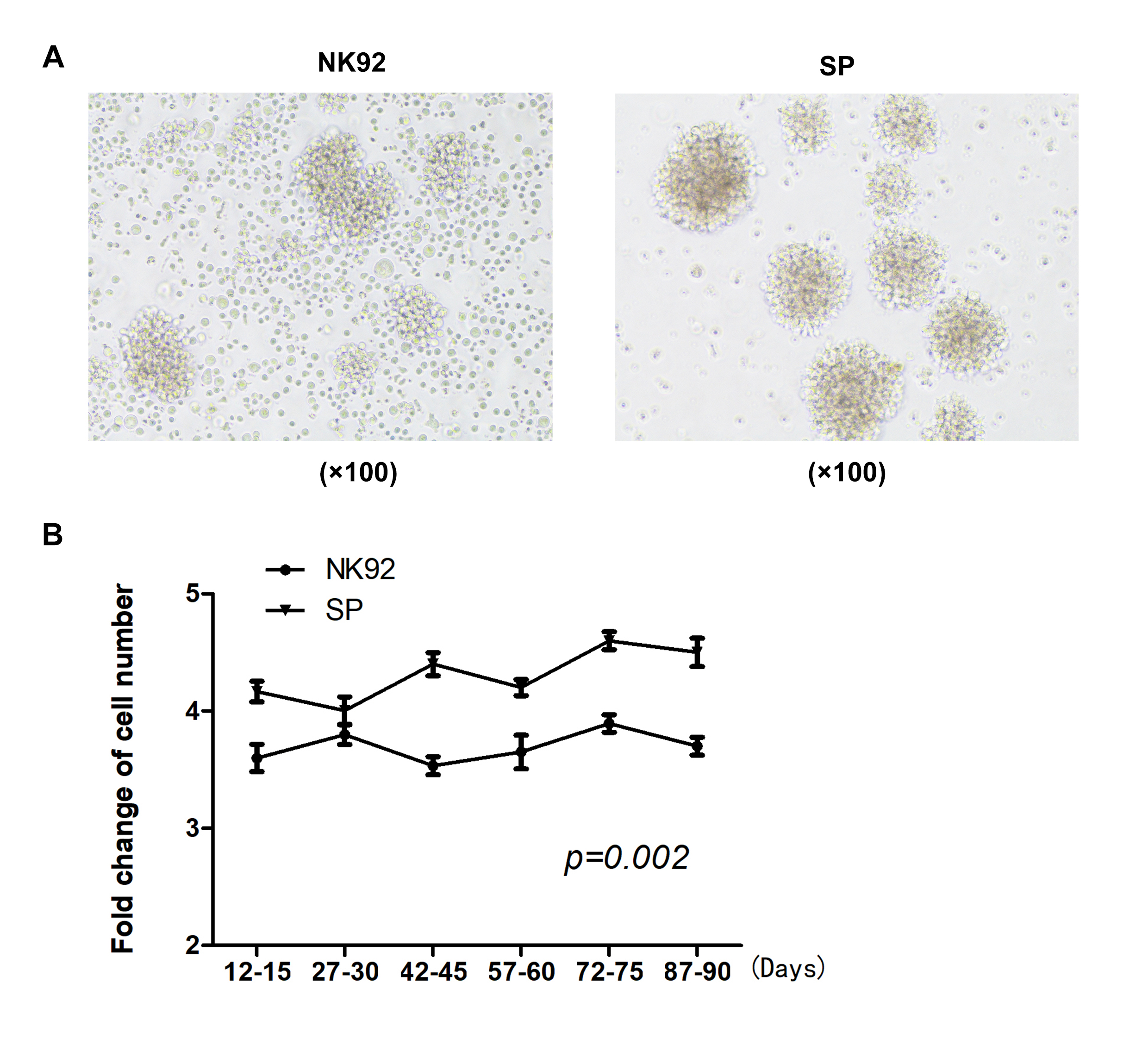

Supplement: Supplementary Figure 2 — Characteristics of SP cells. (A) The observation of NK92 cell line and SP cell line by microscopic (×100). (B) The SP group was cultured without IL-2, and the NK92 group was cultured with 100U/ml IL-2. The 3-day amplification multiple was detected every 15 days and monitored continuously for 3 months. The 3-day proliferation multiple of the cells in the two groups was compared and analyzed. [file Image_2.jpeg]

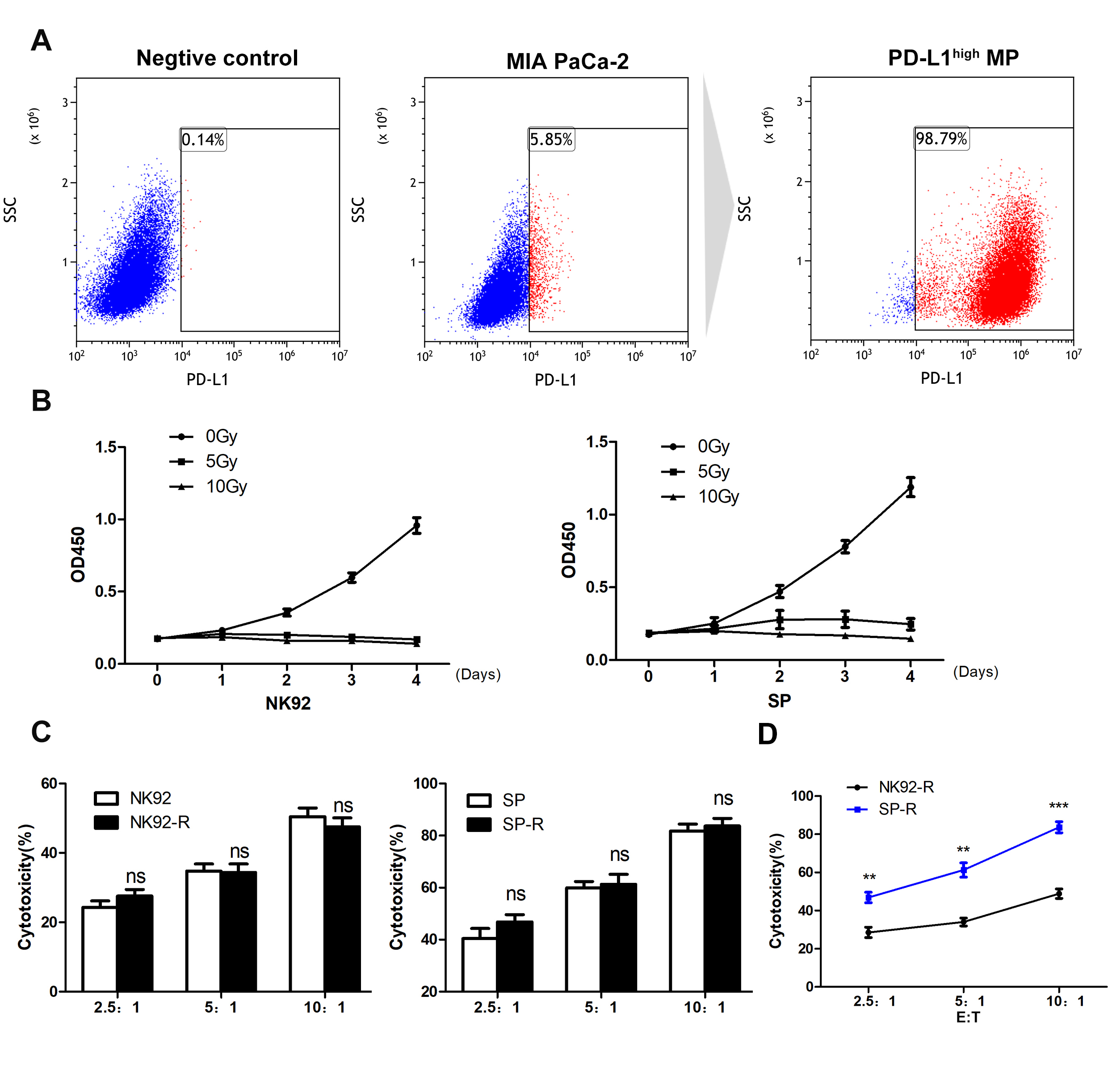

Supplement: Supplementary Figure 3 — Effector and target cell preparation before in vivo experiments. (A) The PD-L1 expression of PD-L1high MP cells was detected by FCM. (B) Detection of the proliferation activity of NK92 and SP cells irradiated with γ-irradiation at different doses. (C) Detection and statistical analysis of the killing effect of NK92 and SP cells on PD-L1high MP before and after 10Gy γ-irradiation (n=3, *P<0.0 5, **P<0.0 1, *P<0.001). (D) Comparation of the killing ability of NK92 cells and SP cells to PD-L1high MP after irradiation. [file Image_3.jpeg]

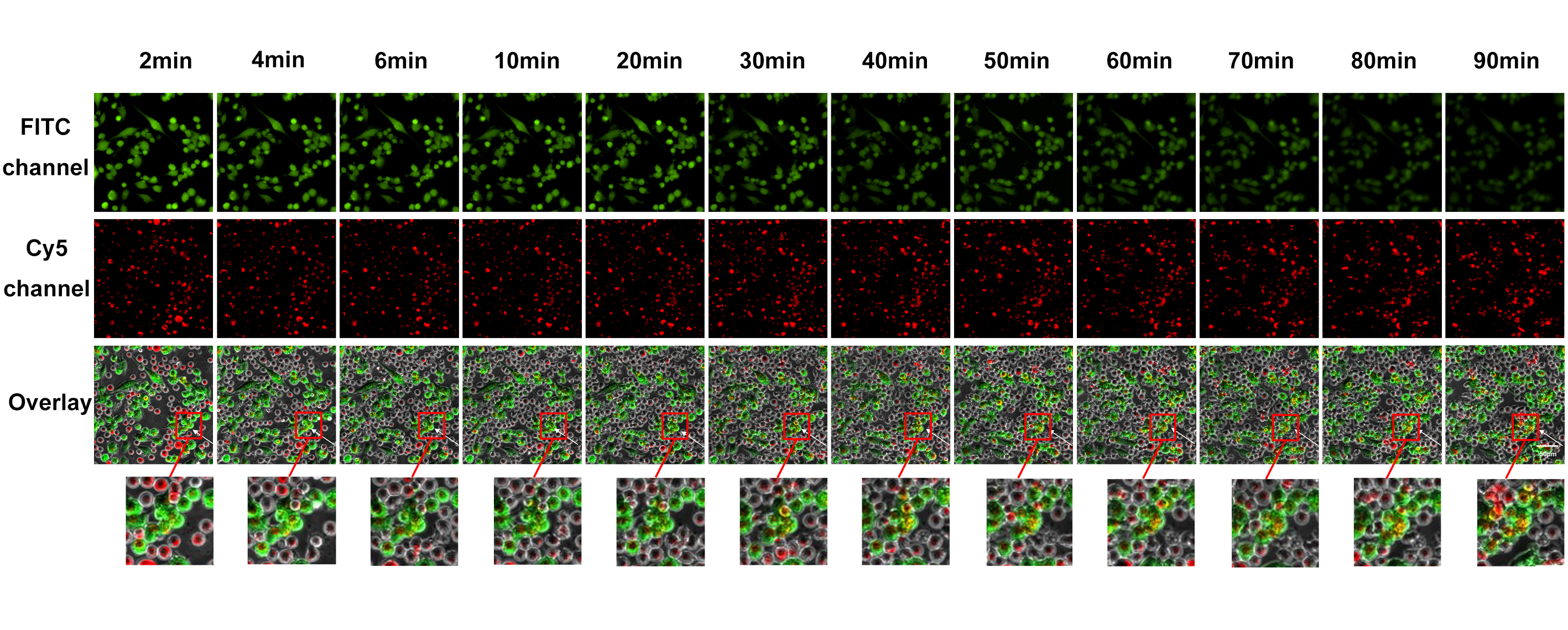

Supplement: Supplementary Figure 4 — Visual and dynamical observation of the delivery process of secretory lysosomes of NK92 cells to PD-L1highMP cells. From left to right are the images from 12 sequential time points of the effect-target cell co-culture experiment. From top to bottom, the first row indicates fields of view under the green fluorescence (FITC) channel, the second row indicates fields of view under the red fluorescence (Cy5) channel, and the third row shows the overlay of the two-fluorescence signals. Dynamically changes can be observed following the co-culture of effector and target cells, and the red box indicates typical degranulation phenomenon, in which NK92 cells delivered secretory lysosomes labeled by Lyso-Tracker Red fluorescent probe to PD-L1highMP cells. [file Image_4.jpeg]
