# Supplementary material for: A Novel Sushi-IL15-PD1 CAR-NK92 Cell Line With Enhanced and PD-L1 Targeted Cytotoxicity Against Pancreatic Cancer Cells
Source: Front Oncol. 2022 Mar 22;12:726985. doi: 10.3389/fonc.2022.726985 (PMC8980464; doi:10.3389/fonc.2022.726985)
Supplement: Supplementary file 5 [file DataSheet_1.doc]

[Signal-peptide]

Amino acid numbering used in the Uniprot database: P01732-CD8A (1-21)

Nucleotide Sequence (63 nt):

Atggccttaccagtgaccgccttgctcctgccgctggccttgctgctccacgccgccaggccg

Amino Acid Sequence (21aa):

MALPVTALLLPLALLLHAARP

BLAST in the Uniprot database


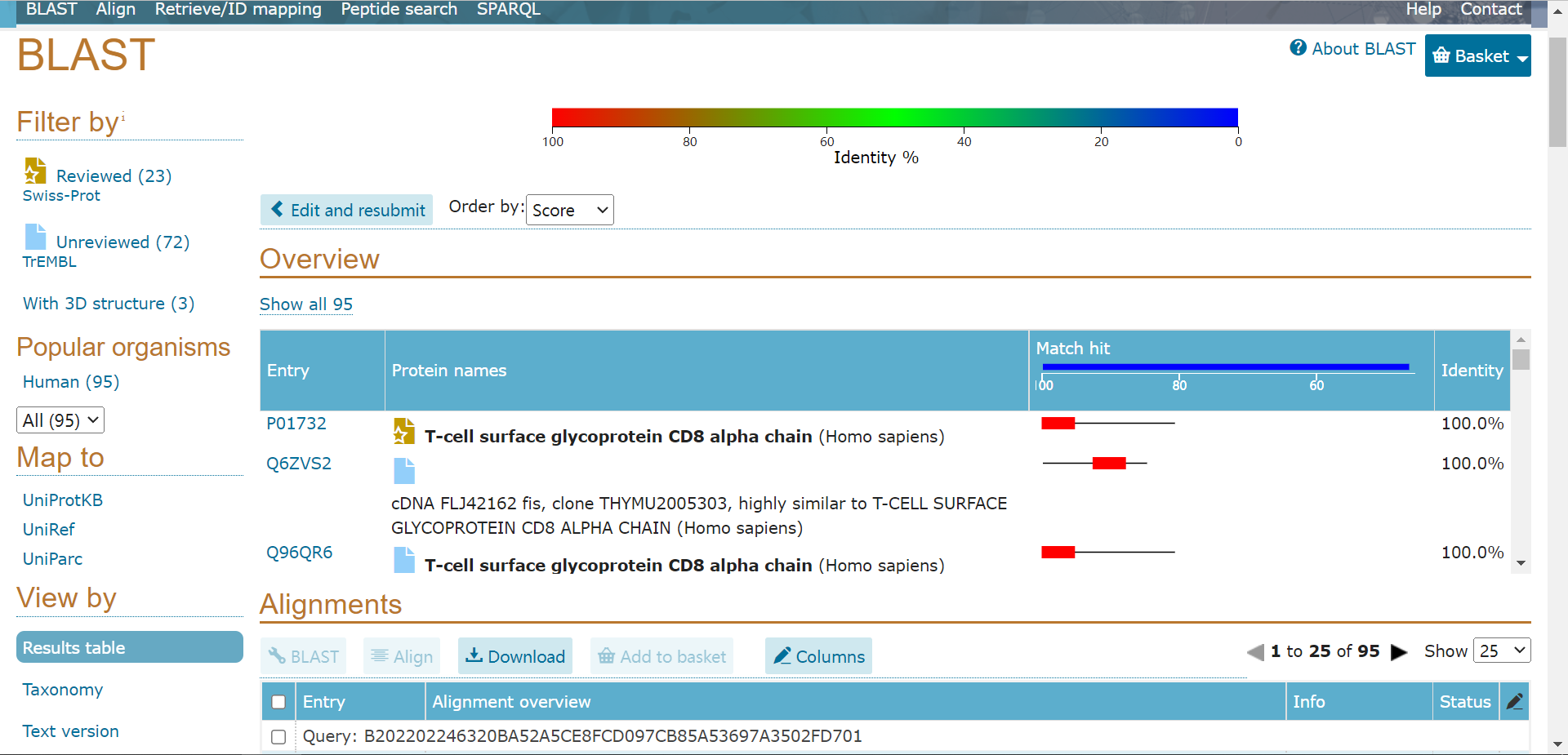


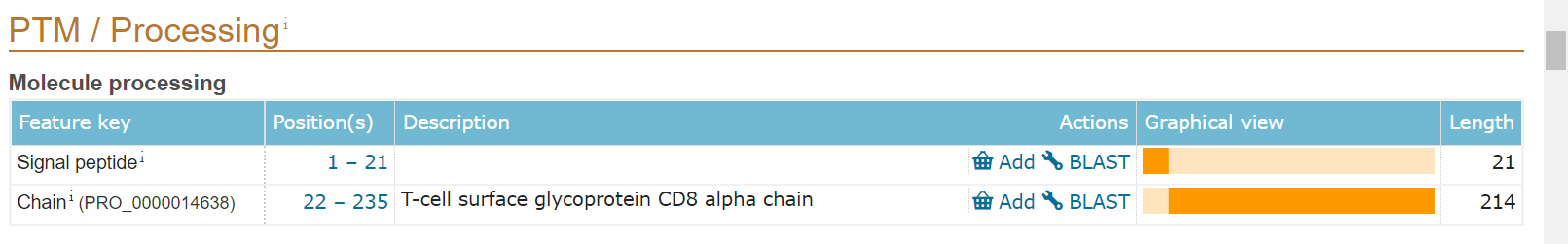


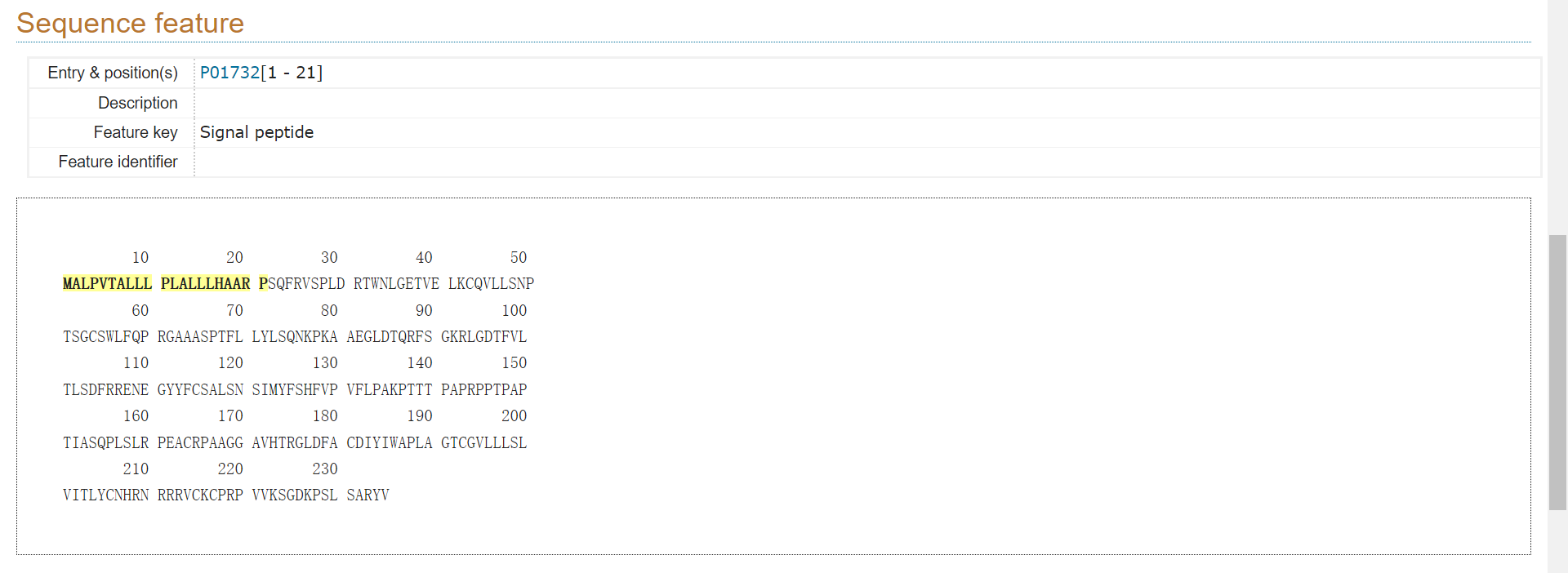


Entry: P01732

>sp|P01732|CD8A_HUMAN T-cell surface glycoprotein CD8 alpha chain OS=Homo sapiens OX=9606 GN=CD8A PE=1 SV=1

MALPVTALLLPLALLLHAARPSQFRVSPLDRTWNLGETVELKCQVLLSNPTSGCSWLFQPRGAAASPTFLLYLSQNKPKAAEGLDTQRFSGKRLGDTFVLTLSDFRRENEGYYFCSALSNSIMYFSHFVPVFLPAKPTTTPAPRPPTPAPTIASQPLSLRPEACRPAAGGAVHTRGLDFACDIYIWAPLAGTCGVLLLSLVITLYCNHRNRRRVCKCPRPVVKSGDKPSLSARYV

[IL15Rα-sushi]

Amino acid numbering used in the Uniprot database: Q13261- IL15RA (31-102)

Nucleotide Sequence (216 nt):

Atcacgtgccctccccccatgtccgtggaacacgcagacatctgggtcaagagctacagcttgtactccagggagcggtacatttgtaactctggtttcaagcgtaaagccggcacgtccagcctgacggagtgcgtgttgaacaaggccacgaatgtcgcccactggacaacccccagtctcaaatgcattagagaccctgccctggttcaccaa

Amino Acid Sequence (72 aa):

ITCPPPMSVEHADIWVKSYSLYSRERYICNSGFKRKAGTSSLTECVLNKATNVAHWTTPSLKCIRDPALVHQ

BLAST in the Uniprot database


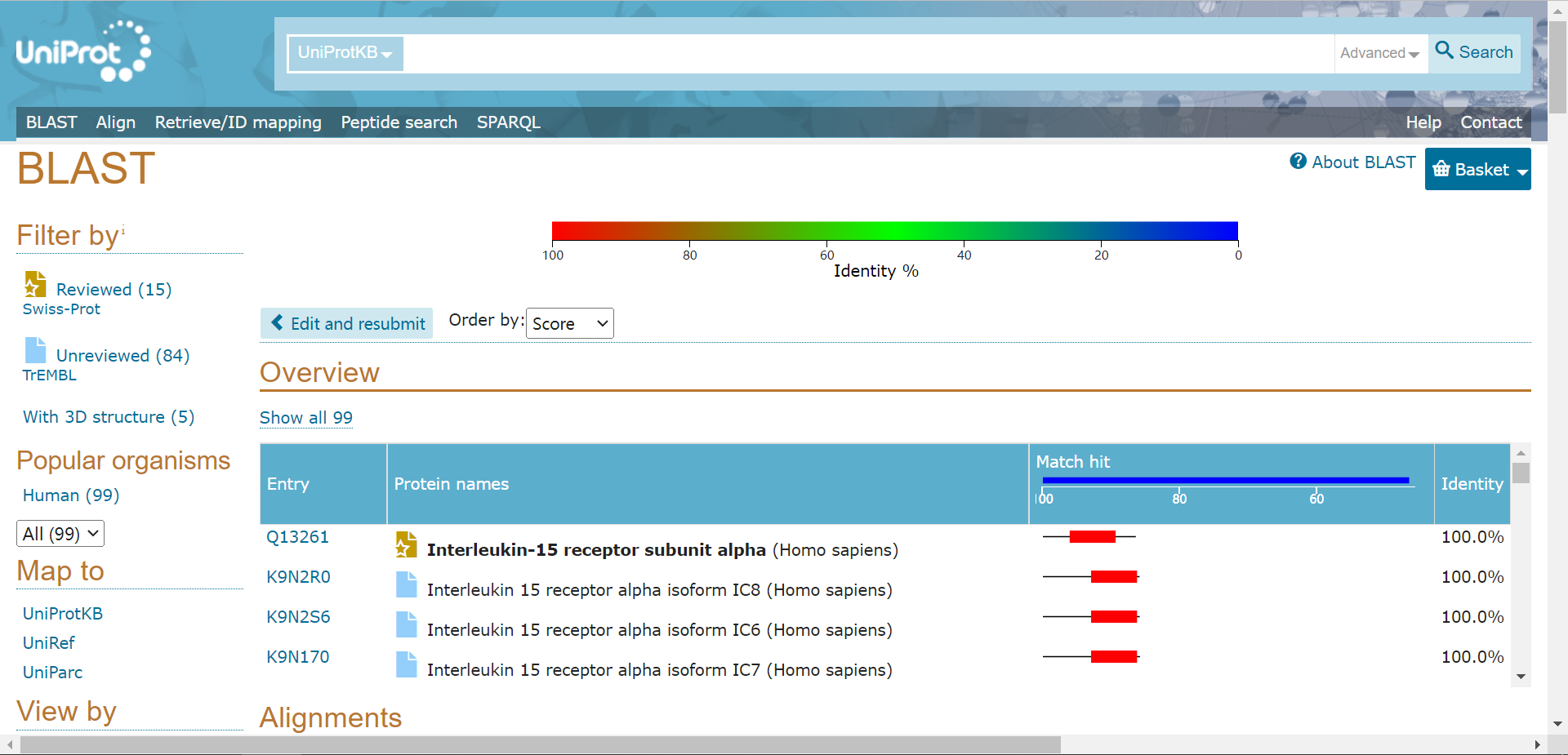


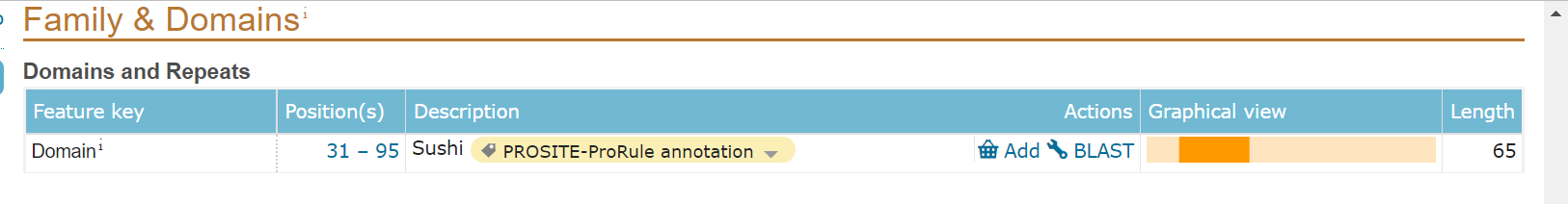


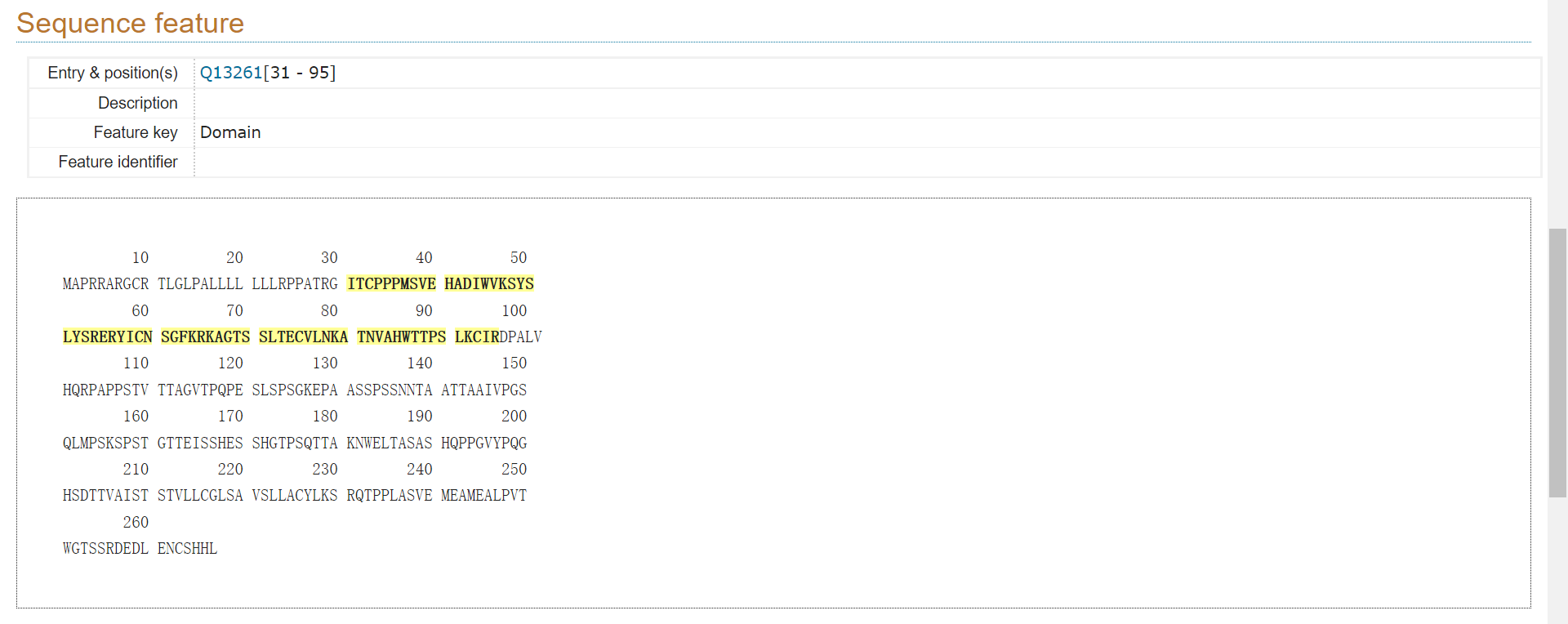


Entry: Q13261

>sp|Q13261|I15RA_HUMAN Interleukin-15 receptor subunit alpha OS=Homo sapiens OX=9606 GN=IL15RA PE=1 SV=1

MAPRRARGCRTLGLPALLLLLLLRPPATRGITCPPPMSVEHADIWVKSYSLYSRERYICNSGFKRKAGTSSLTECVLNKATNVAHWTTPSLKCIRDPALVHQRPAPPSTVTTAGVTPQPESLSPSGKEPAASSPSSNNTAATTAAIVPGSQLMPSKSPSTGTTEISSHESSHGTPSQTTAKNWELTASASHQPPGVYPQGHSDTTVAISTSTVLLCGLSAVSLLACYLKSRQTPPLASVEMEAMEALPVTWGTSSRDEDLENCSHHL

[CD8a-TMD(transmembrane domain)]

Amino acid numbering used in the Uniprot database: P01732-CD8A (138-206)

Nucleotide Sequence (207 nt):

accacgacgccagcgccgcgaccaccaacaccggcgcccaccatcgcgtcgcagcccctgtccctgcgcccagaggcgtgccggccagcggcggggggcgcagtgcacacgagggggctggacttcgcctgtgacatctacatctgggcgcccttggccgggacttgtggggtccttctcctgtcactggttatcaccctttactgc

Amino Acid Sequence (69 aa):

TTTPAPRPPTPAPTIASQPLSLRPEACRPAAGGAVHTRGLDFACDIYIWAPLAGTCGVLLLSLVITLYC

BLAST in the Uniprot database


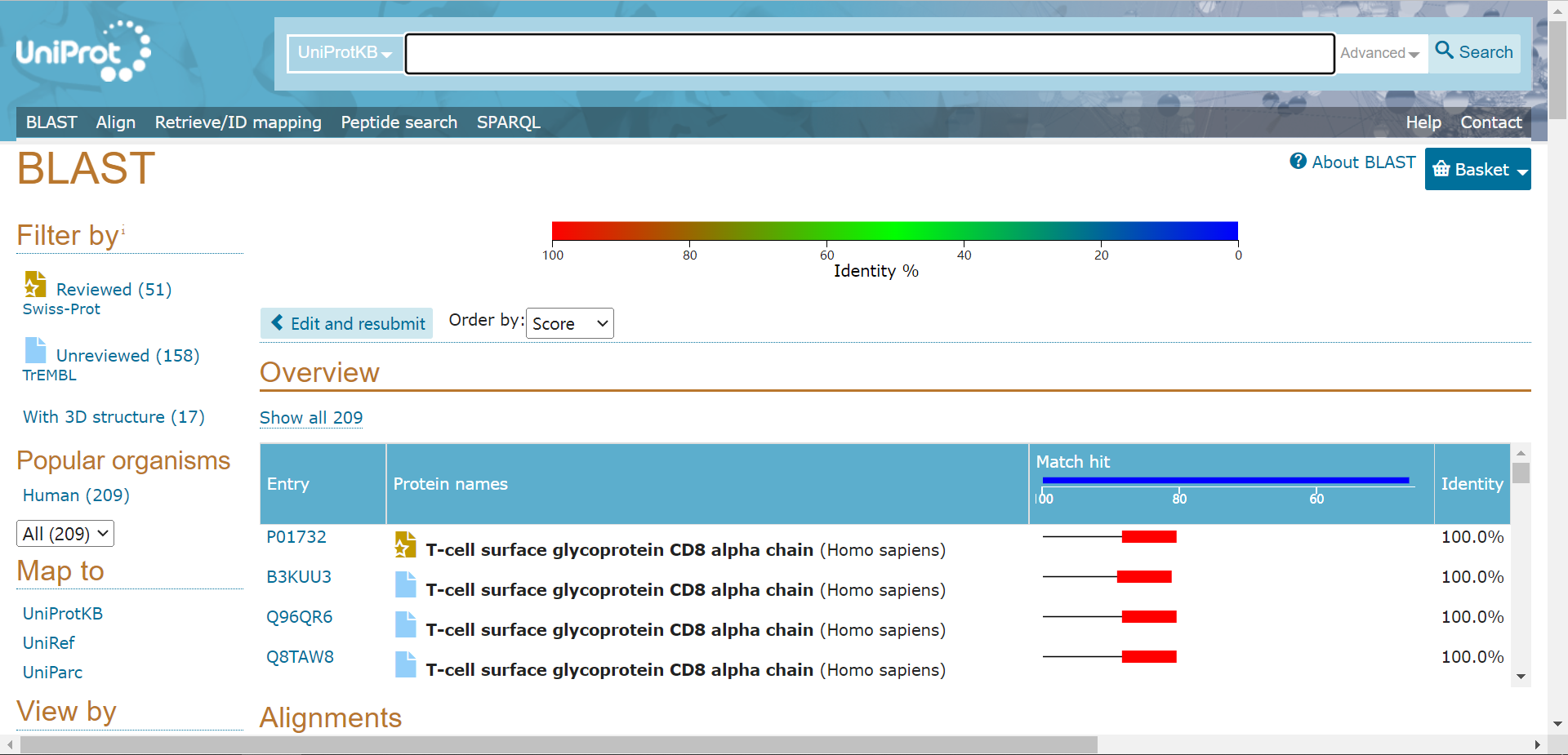


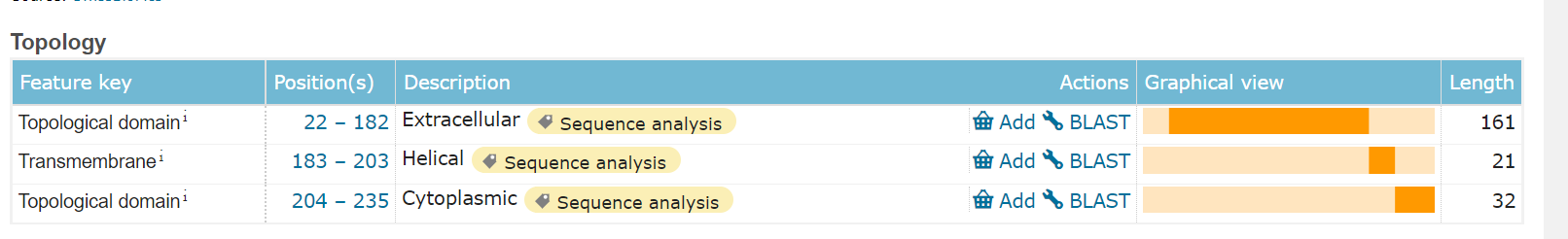


Entry: P01732

>sp|P01732|CD8A_HUMAN T-cell surface glycoprotein CD8 alpha chain OS=Homo sapiens OX=9606 GN=CD8A PE=1 SV=1

MALPVTALLLPLALLLHAARPSQFRVSPLDRTWNLGETVELKCQVLLSNPTSGCSWLFQPRGAAASPTFLLYLSQNKPKAAEGLDTQRFSGKRLGDTFVLTLSDFRRENEGYYFCSALSNSIMYFSHFVPVFLPAKPTTTPAPRPPTPAPTIASQPLSLRPEACRPAAGGAVHTRGLDFACDIYIWAPLAGTCGVLLLSLVITLYCNHRNRRRVCKCPRPVVKSGDKPSLSARYV

[4-1BB]

Amino acid numbering used in the Uniprot database: Q07011- TNR9 (214-255)

Nucleotide Sequence (126 nt):

aaacggggcagaaagaaactcctgtatatattcaaacaaccatttatgagaccagtacaaactactcaagaggaagatggctgtagctgccgatttccagaagaagaagaaggaggatgtgaactg

Amino Acid Sequence (42 aa):

KRGRKKLLYIFKQPFMRPVQTTQEEDGCSCRFPEEEEGGCEL

BLAST in the Uniprot database


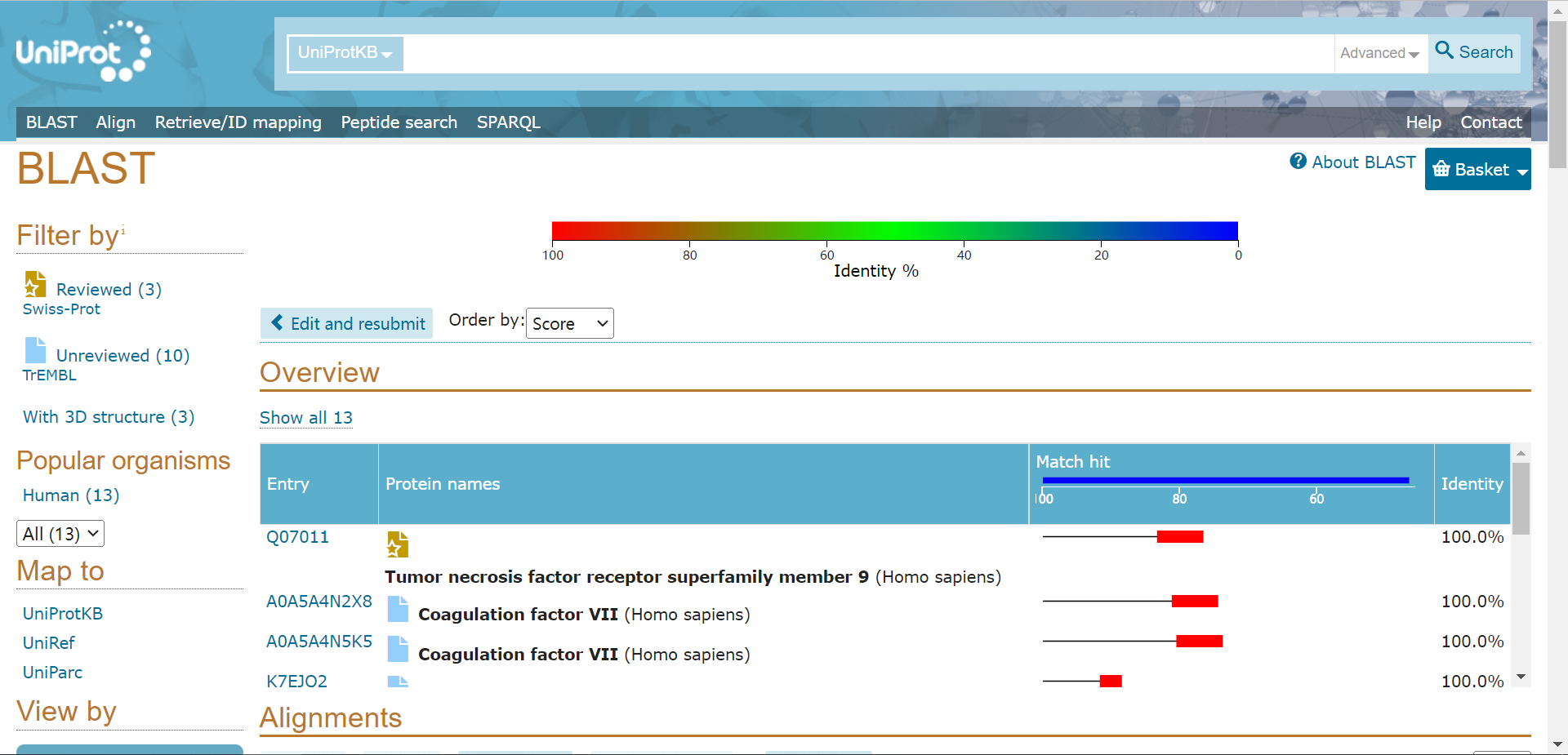


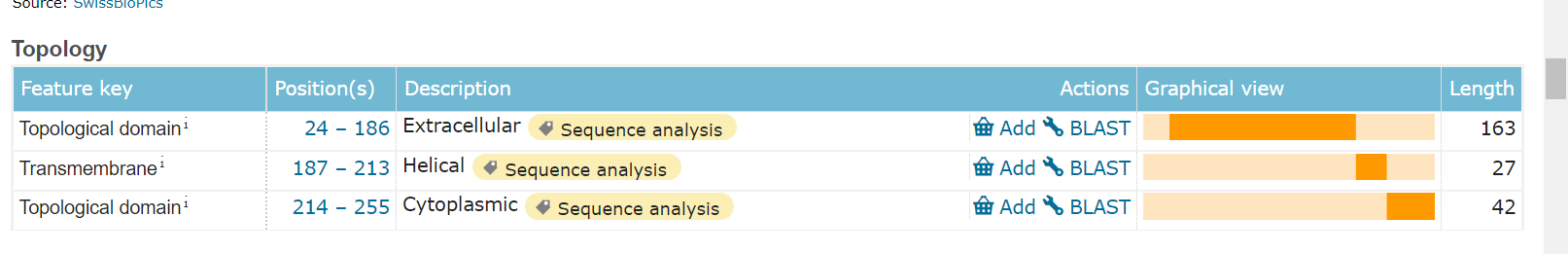


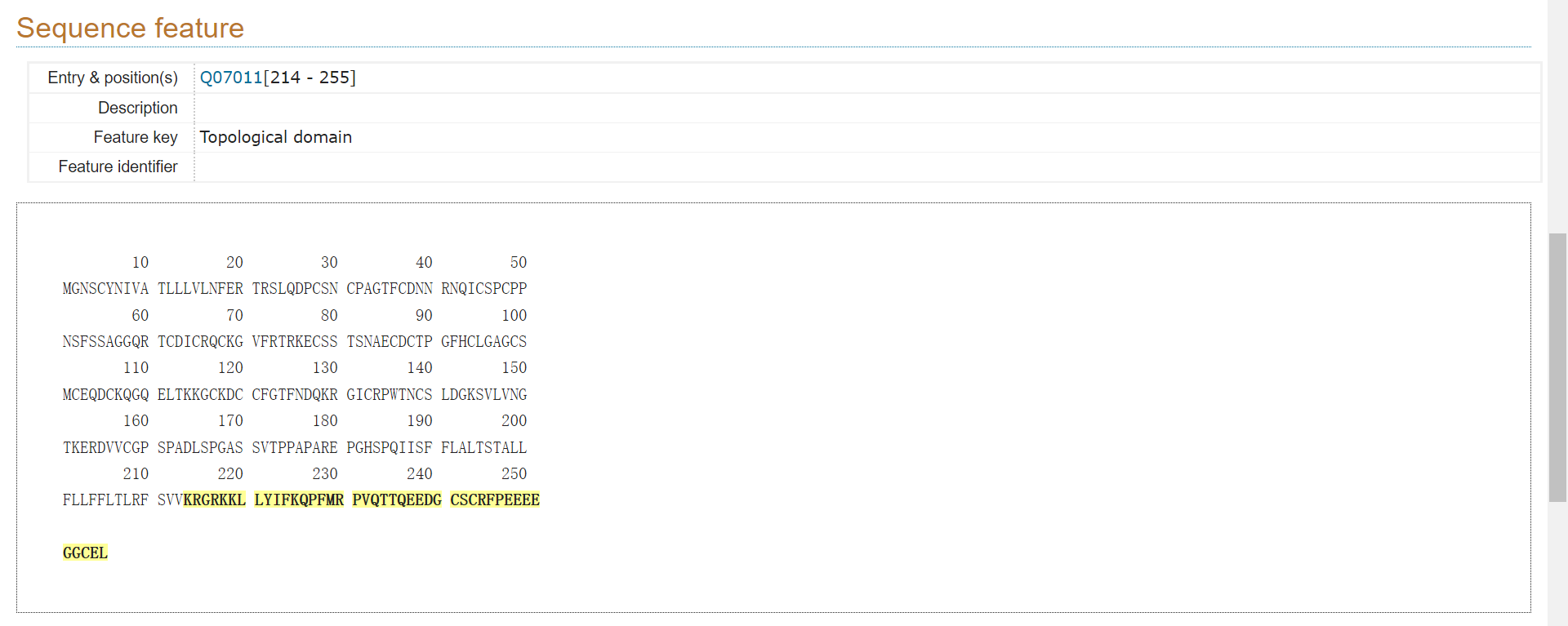


Entry: Q07011

>sp|Q07011|TNR9_HUMAN Tumor necrosis factor receptor superfamily member 9 OS=Homo sapiens OX=9606 GN=TNFRSF9 PE=1 SV=1

MGNSCYNIVATLLLVLNFERTRSLQDPCSNCPAGTFCDNNRNQICSPCPPNSFSSAGGQRTCDICRQCKGVFRTRKECSSTSNAECDCTPGFHCLGAGCSMCEQDCKQGQELTKKGCKDCCFGTFNDQKRGICRPWTNCSLDGKSVLVNGTKERDVVCGPSPADLSPGASSVTPPAPAREPGHSPQIISFFLALTSTALLFLLFFLTLRFSVVKRGRKKLLYIFKQPFMRPVQTTQEEDGCSCRFPEEEEGGCEL

[CD3ζ]

Amino acid numbering used in the Uniprot database: P20963- CD3Z (52-164)

Nucleotide Sequence (336 nt):

Agagtgaagttcagcaggagcgcagacgcccccgcgtacaagcagggccagaaccagctctataacgaactcaatctaggacgaagagaggagtacgatgttttggacaagagacgtggccgggaccctgagatggggggaaagccgagaaggaagaaccctcaggaaggcctgtacaatgaactgcagaaagataagatggcggaggcctacagtgagattgggatgaaaggcgagcggcggaggggcaaggggcacgatggcctttaccagggtctcagtacagccaccaaggacacctacgacgcccttcacatgcaggccctgccccctcgc

Amino Acid Sequence (112 aa):

RVKFSRSADAPAYKQGQNQLYNELNLGRREEYDVLDKRRGRDPEMGGKPRRKNPQEGLYNELQKDKMAEAYSEIGMKGERRRGKGHDGLYQGLSTATKDTYDALHMQALPPR

BLAST in the Uniprot database


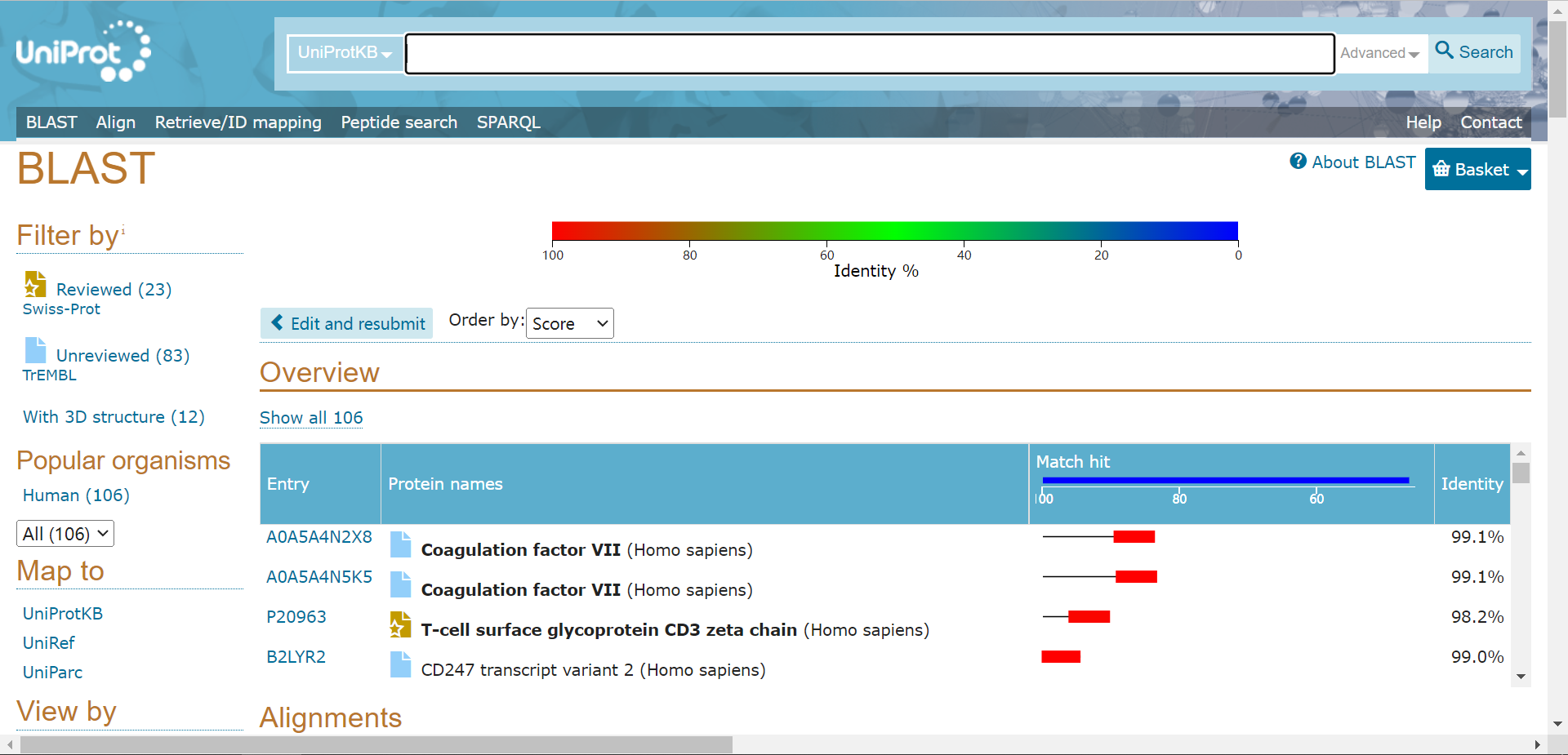


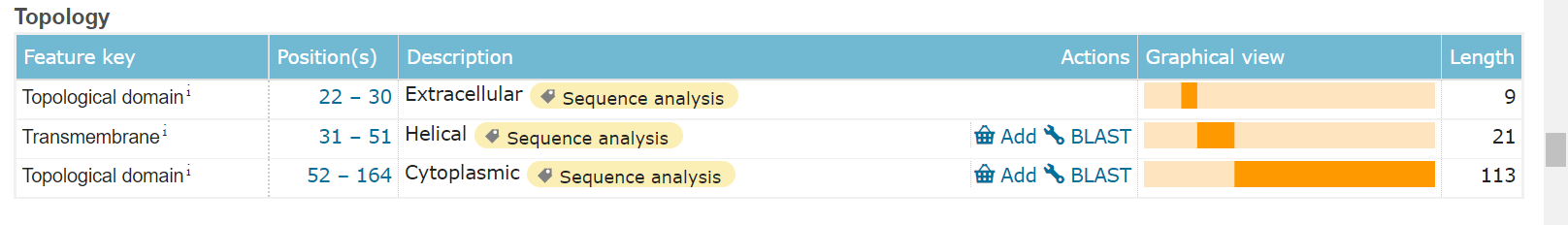


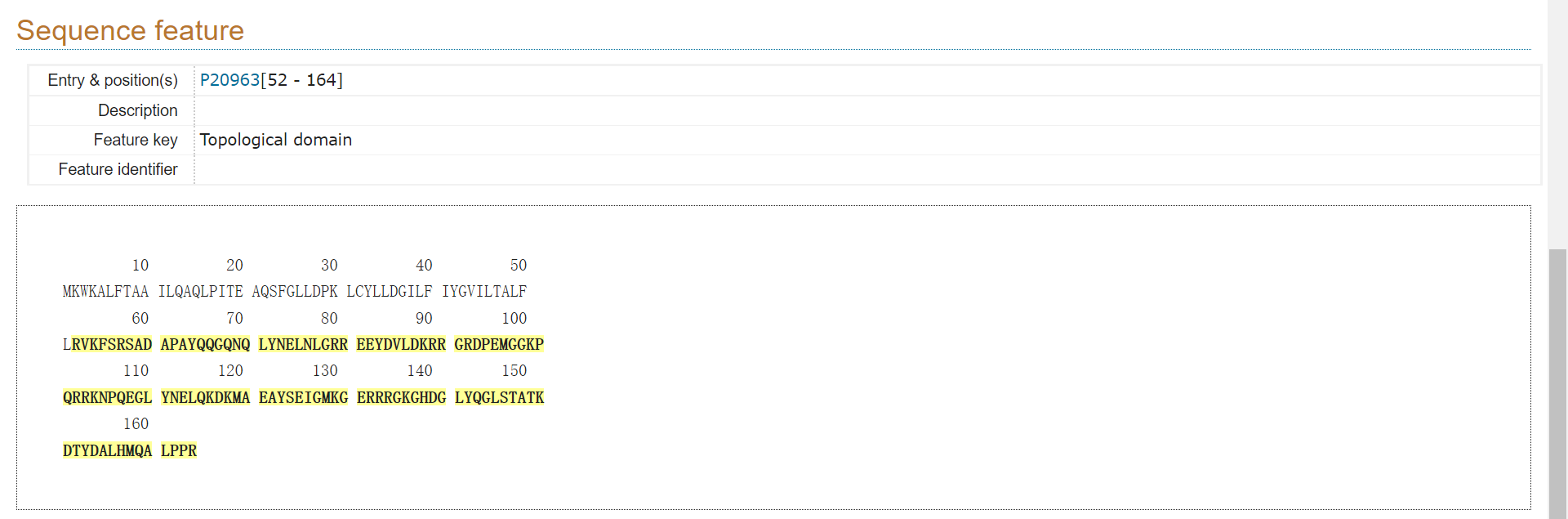


Entry: P20963

>sp|P20963|CD3Z_HUMAN T-cell surface glycoprotein CD3 zeta chain OS=Homo sapiens OX=9606 GN=CD247 PE=1 SV=2

MKWKALFTAAILQAQLPITEAQSFGLLDPKLCYLLDGILFIYGVILTALFLRVKFSRSADAPAYQQGQNQLYNELNLGRREEYDVLDKRRGRDPEMGGKPQRRKNPQEGLYNELQKDKMAEAYSEIGMKGERRRGKGHDGLYQGLSTATKDTYDALHMQALPPR

[P2A]

Nucleotide Sequence (66 nt):

GGAAGCGGCGCCACCAACTTCTCCCTGCTGAAGCAAGCTGGCGACGTGGAAGAGAACCCCGGCCCC

Amino Acid Sequence (22 aa):

GSGATNFSLLKQAGDVEENPGP

[PD1-Extracellular Domain]

Amino acid numbering used in the Uniprot database: Q15116- PDCD1 (21-170)

Nucleotide Sequence (450 nt):

Ccaggatggttcttagactccccagacaggccctggaacccccccaccttctccccagccctgctcgtggtgaccgaaggggacaacgccaccttcacctgcagcttctccaacacatcggagagcttcgtgctaaactggtaccgcatgagccccagcaaccagacggacaagctggccgccttccccgaggaccgcagccagcccggccaggactgccgcttccgtgtcacacaactgcccaacgggcgtgacttccacatgagcgtggtcagggcccggcgcaatgacagcggcacctacctctgtggggccatctccctggcccccaaggcgcagatcaaagagagcctgcgggcagagctcagggtgacagagagaagggcagaagtgcccacagcccaccccagcccctcacccaggccagccggccagttccaaaccctggtg

Amino Acid Sequence (150 aa):

PGWFLDSPDRPWNPPTFSPALLVVTEGDNATFTCSFSNTSESFVLNWYRMSPSNQTDKLAAFPEDRSQPGQDCRFRVTQLPNGRDFHMSVVRARRNDSGTYLCGAISLAPKAQIKESLRAELRVTERRAEVPTAHPSPSPRPAGQFQTLV

BLAST in the Uniprot database


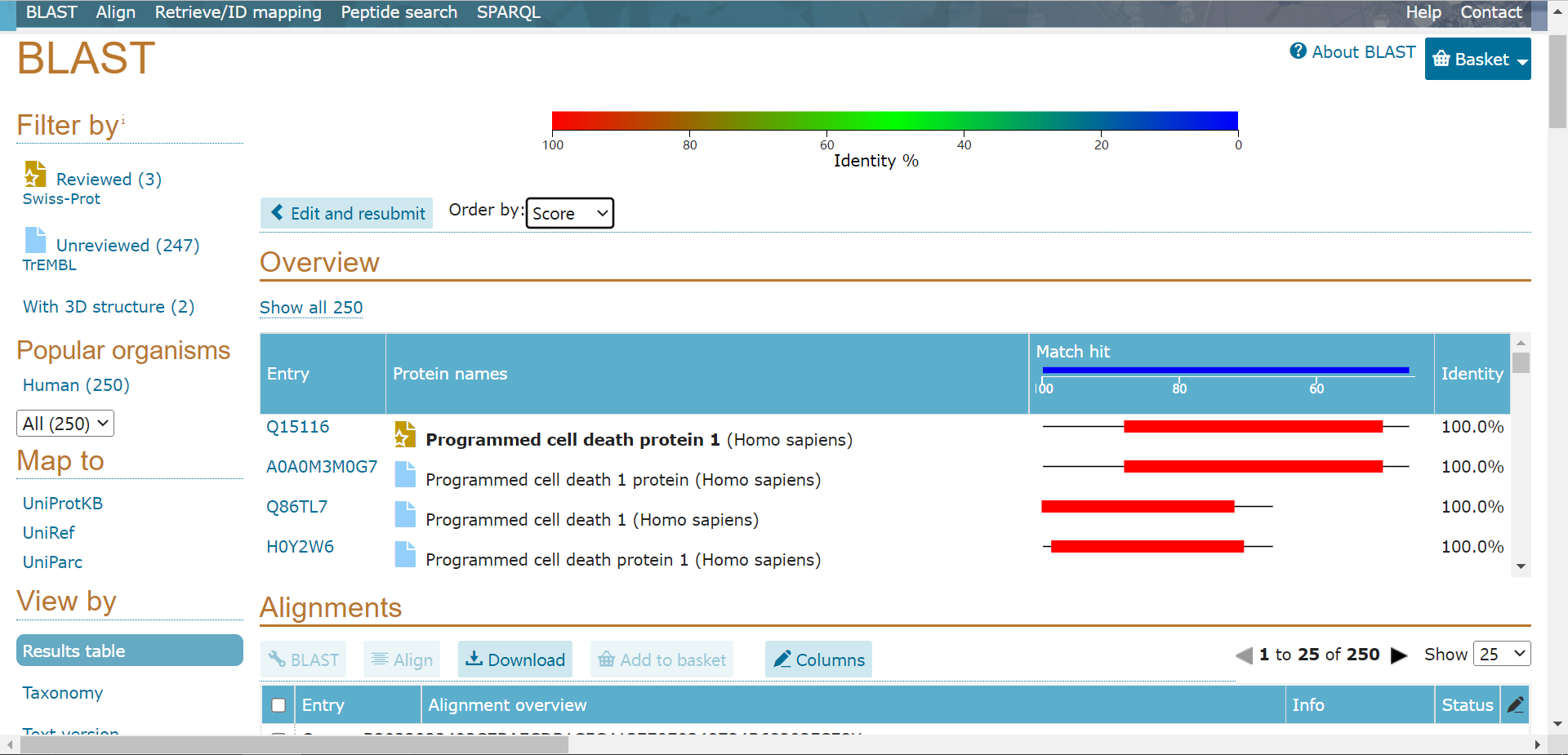


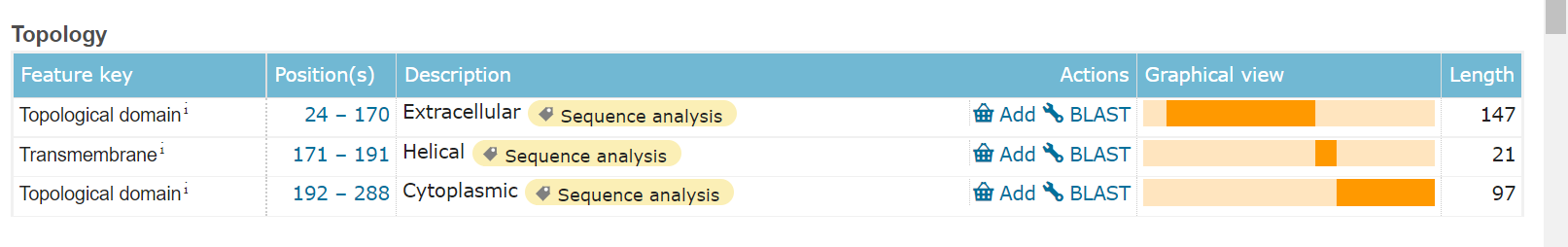


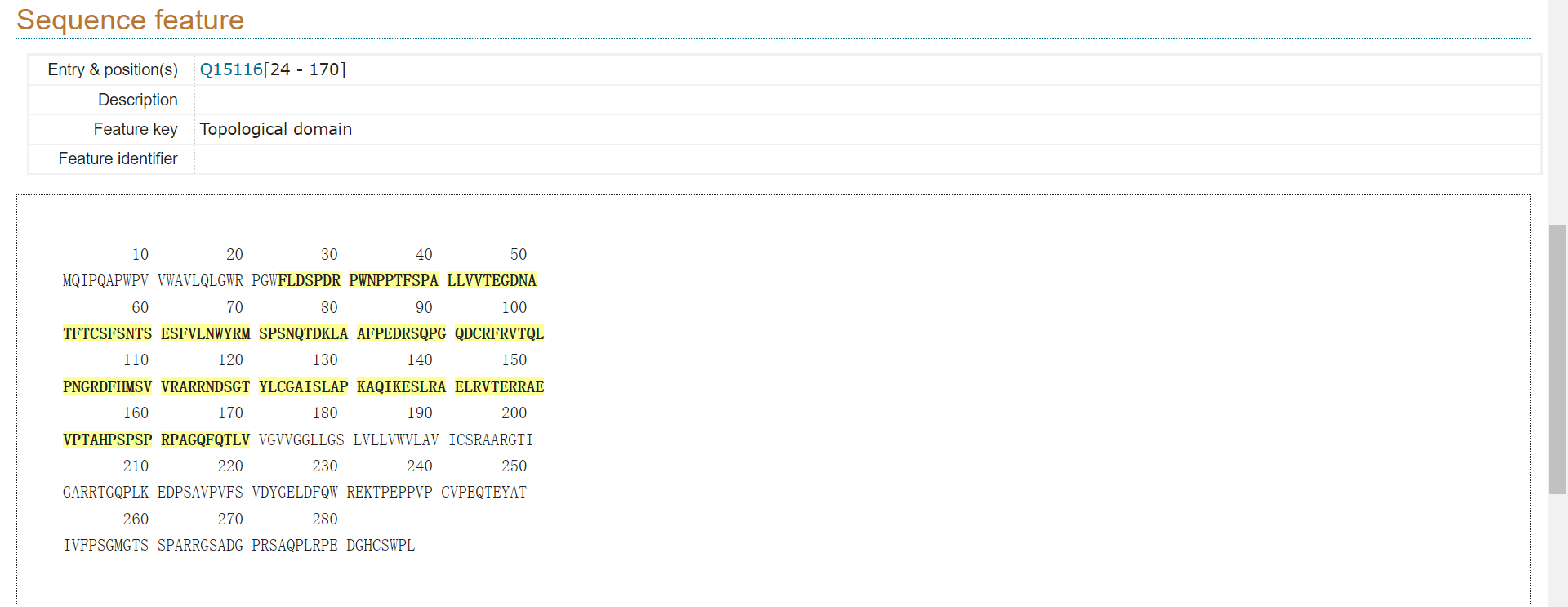


Entry: Q15116

>sp|Q15116|PDCD1_HUMAN Programmed cell death protein 1 OS=Homo sapiens OX=9606 GN=PDCD1 PE=1 SV=3

MQIPQAPWPVVWAVLQLGWRPGWFLDSPDRPWNPPTFSPALLVVTEGDNATFTCSFSNTSESFVLNWYRMSPSNQTDKLAAFPEDRSQPGQDCRFRVTQLPNGRDFHMSVVRARRNDSGTYLCGAISLAPKAQIKESLRAELRVTERRAEVPTAHPSPSPRPAGQFQTLVVGVVGGLLGSLVLLVWVLAVICSRAARGTIGARRTGQPLKEDPSAVPVFSVDYGELDFQWREKTPEPPVPCVPEQTEYATIVFPSGMGTSSPARRGSADGPRSAQPLRPEDGHCSWPL

[linker]

Nucleotide Sequence ( 15nt):

GGCGGAGGAGGCTCC

Amino Acid Sequence ( 5aa):

GGGGS

[IL15]

Amino acid numbering used in the Uniprot database: P40933- IL15 (49-162)

Nucleotide Sequence (342 nt):

AACTGGGTGAACGTCATCTCCGACCTCAAGAAGATCGAGGACCTGATCCAGAGCATGCACATCGACGCCACCCTGTATACCGAGAGCGACGTGCACCCCTCCTGTAAAGTGACCGCCATGAAGTGCTTCCTGCTGGAGCTGCAGGTGATCAGCCTGGAGAGCGGCGACGCCAGCATCCATGACACCGTGGAGAACCTGATCATCCTGGCCAATAACAGCCTGAGCTCCAACGGCAACGTGACCGAGAGCGGCTGCAAGGAATGCGAGGAGCTGGAGGAAAAGAACATTAAGGAGTTCCTGCAGAGCTTCGTCCACATCGTGCAGATGTTCATTAACACCTCCTAATGA

Amino Acid Sequence (114 aa):

NWVNVISDLKKIEDLIQSMHIDATLYTESDVHPSCKVTAMKCFLLELQVISLESGDASIHDTVENLIILANNSLSSNGNVTESGCKECEELEEKNIKEFLQSFVHIVQMFINTS**

BLAST in the Uniprot database


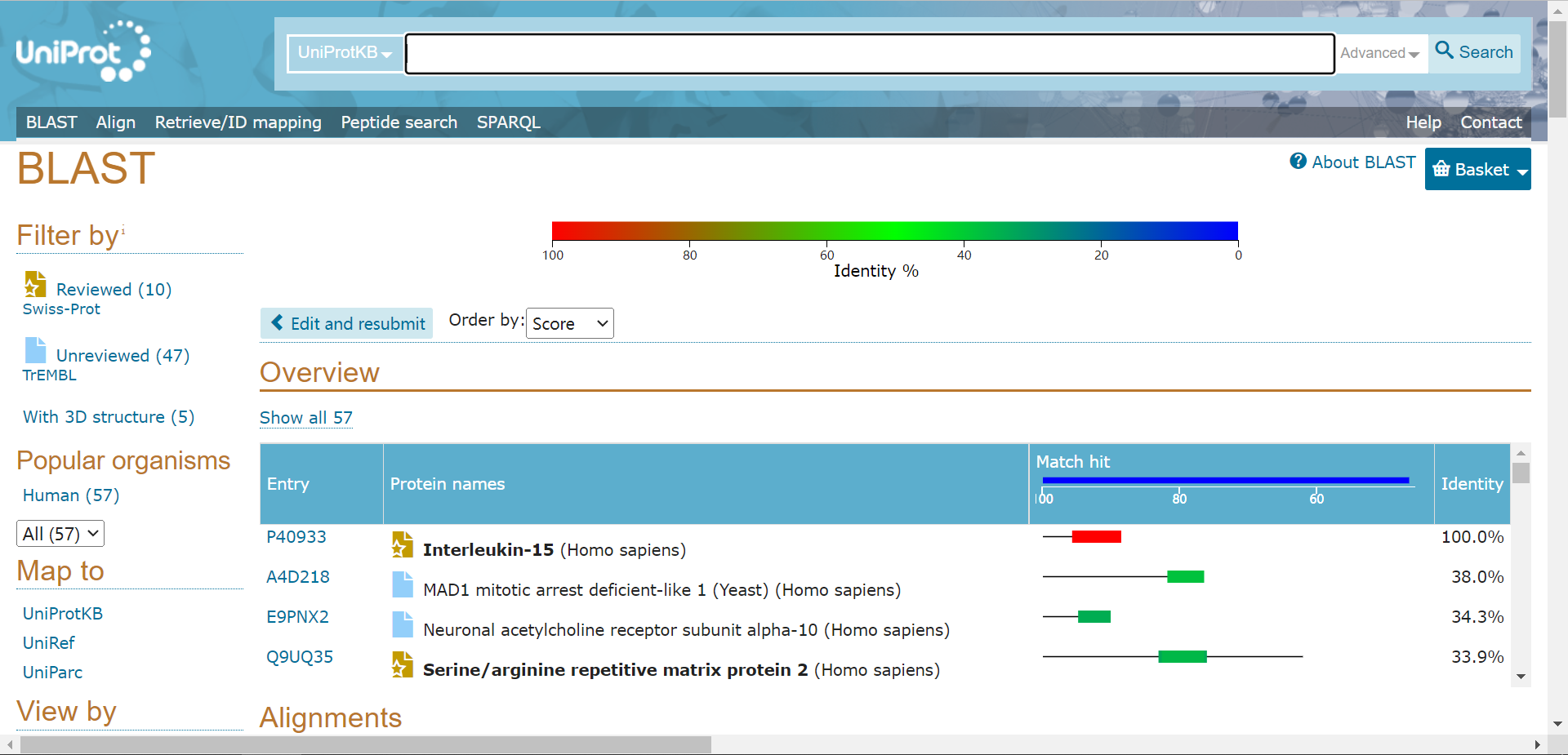


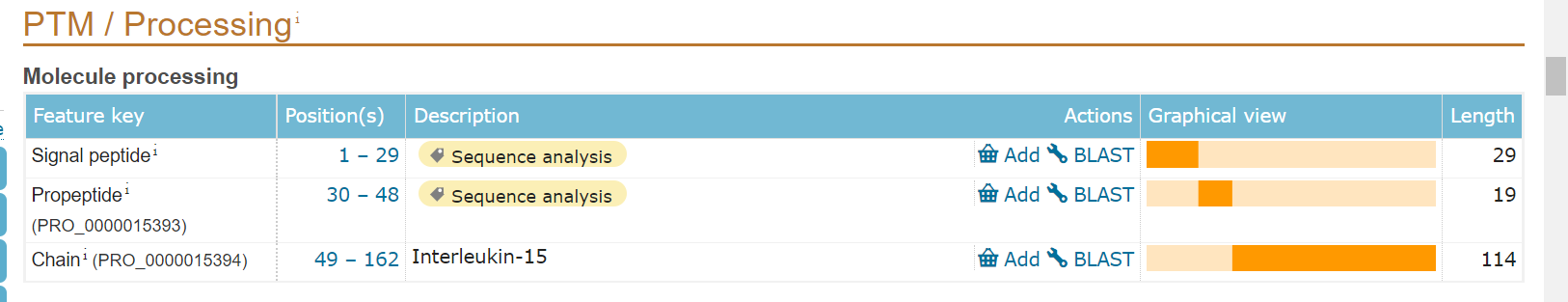


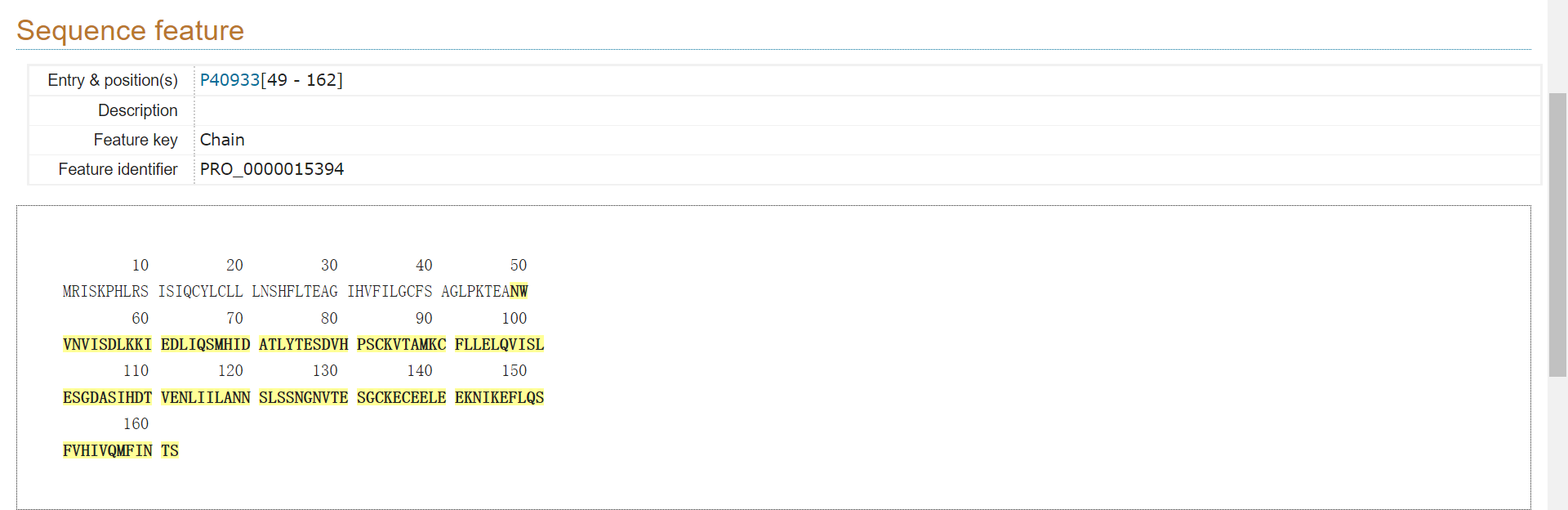


Entry: P40933

>sp|P40933|IL15_HUMAN Interleukin-15 OS=Homo sapiens OX=9606 GN=IL15 PE=1 SV=1

MRISKPHLRSISIQCYLCLLLNSHFLTEAGIHVFILGCFSAGLPKTEANWVNVISDLKKIEDLIQSMHIDATLYTESDVHPSCKVTAMKCFLLELQVISLESGDASIHDTVENLIILANNSLSSNGNVTESGCKECEELEEKNIKEFLQSFVHIVQMFINTS
