# Supplementary material for: A Novel Sushi-IL15-PD1 CAR-NK92 Cell Line With Enhanced and PD-L1 Targeted Cytotoxicity Against Pancreatic Cancer Cells
Source: Front Oncol. 2022 Mar 22;12:726985. doi: 10.3389/fonc.2022.726985 (PMC8980464; doi:10.3389/fonc.2022.726985)
Supplement: Supplementary file 6 [file Table_1.docx]

**Supplemental: Table 1. Antibody information.**

| Source | Antibody |
| --- | --- |
| ebioscience | Granzyme B (GB11, PE, GRB04), CD56 (CMSSB, APC, 17-0567-42) |
| BD Biosciences | NKG2D (1D11, PE, 561815), CD226 (DX11, FITC, 559788), LFA-1 (TS1/22, PE), PD-L1 (MIH1, PE, 557924), PD-1 (EH12, BV510, 563076), |
| Biolegend | TNF-α (MAb11, APC , 502912)，IFN-γ (B27, PC5.5, 506528), TRAIL (RIK-2, PE, 308206), CD107a (H4A3, BV421, 328626), Fas-L (NKO-1, BV421, 306412), Perforin (δG9, BV510, Perforin), NKp30 (P30-15, APC, 325226) |
